# Exciton-assisted electron tunnelling in van der Waals heterostructures

**DOI:** 10.1038/s41563-023-01556-7

**Published:** 2023-06-26

**Authors:** Lujun Wang, Sotirios Papadopoulos, Fadil Iyikanat, Jian Zhang, Jing Huang, Takashi Taniguchi, Kenji Watanabe, Michel Calame, Mickael L. Perrin, F. Javier García de Abajo, Lukas Novotny

**Affiliations:** 1 Photonics Laboratory, ETH Zürich, Zürich, Switzerland; 2 Institut de Ciències Fotòniques (ICFO), The Barcelona Institute of Science and Technology, Castelldefels, Spain; 3 Transport at Nanoscale Interfaces Laboratory, Empa, Swiss Federal Laboratories for Materials Science and Technology, Dübendorf, Switzerland; 4 International Center for Materials Nanoarchitectonics, National Institute for Materials Science, Tsukuba, Japan; 5 Research Center for Functional Materials, National Institute for Materials Science, Tsukuba, Japan; 6 Department of Physics, University of Basel, Basel, Switzerland; 7 Swiss Nanoscience Institute, University of Basel, Basel, Switzerland; 8 Department of Information Technology and Electrical Engineering, ETH Zürich, Zürich, Switzerland; 9 Quantum Center, ETH Zürich, Zürich, Switzerland; 10 Institució Catalana de Recerca i Estudis Avançats (ICREA), Barcelona, Spain

**Keywords:** Two-dimensional materials, Electronic properties and materials

## Abstract

The control of elastic and inelastic electron tunnelling relies on materials with well-defined interfaces. Two-dimensional van der Waals materials are an excellent platform for such studies. Signatures of acoustic phonons and defect states have been observed in current-to-voltage measurements. These features can be explained by direct electron–phonon or electron–defect interactions. Here we use a tunnelling process that involves excitons in transition metal dichalcogenides (TMDs). We study tunnel junctions consisting of graphene and gold electrodes separated by hexagonal boron nitride with an adjacent TMD monolayer and observe prominent resonant features in current-to-voltage measurements appearing at bias voltages that correspond to TMD exciton energies. By placing the TMD outside of the tunnelling pathway, we demonstrate that this tunnelling process does not require any charge injection into the TMD. The appearance of such optical modes in electrical transport introduces additional functionality towards van der Waals material–based optoelectronic devices.

## Abstract (web summary)

The authors demonstrate exciton-assisted resonant electron tunnelling in van der Waals heterostructure tunnel junctions. Their study elucidates tunnelling mechanisms involving either indirect or direct excitons in the absence of charge injection and reveals excitonic light emission driven by inelastic electron tunnelling.

## Main

The isolation of two-dimensional (2D) crystals combined with advances in fabrication techniques has enabled the realization of new types of materials, known as van der Waals heterostructures, in which different atomic layers are assembled together in a desired sequence^1^. Tailored heterostructures comprising graphene, hexagonal boron nitride (hBN), TMDs and other 2D materials are currently designed to display properties that are absent in the individual constituents, thus providing a platform for fundamental studies^2–7^ and novel device applications^8–10^. In this respect, tunnel junctions with different material combinations form an interesting system for investigating electron tunnelling processes. Previous experiments have shown phonon-assisted resonant electron tunnelling in metal–insulator junctions^11^, in conventional semiconductor heterostructures^12^ and in graphene-based systems^13–16^. Similarly, exciton-assisted resonant tunnelling has been observed in conventional semiconductor quantum wells^17,18^. Plasmon-assisted resonant tunnelling has been investigated in metallic quantum well structures hosting silver nanorods^19^ and graphene-based structures^20^. Furthermore, evidence for defect-assisted resonant tunnelling has been observed in hBN-based junctions^21^.

Here we demonstrate exciton-assisted resonant electron tunnelling in van der Waals tunnel junctions. Our electron transport measurements reveal distinct resonant peaks that coincide in energy with TMD excitons. We investigate the current-to-voltage (*I*−*V*) characteristics of TMD/graphene/hBN/Au tunnel junctions and compare them with TMD-free reference structures. Resonances observed in differential conductance (d*I*/d*V*) measurements agree with TMD exciton energies despite the fact that the TMD is placed outside the electron tunnelling pathway. These resonances can be explained by a one-step process involving indirect excitons and a two-step process involving both phonons and direct excitons. Both of these processes conserve energy and in-plane momentum. Owing to the large exciton binding energies of TMD monolayers^22,23^, such as WS_2_, MoS_2_, WSe_2_ and MoSe_2_, the resonant features can be observed at room temperature. While exciton–phonon interactions in TMDs have been investigated by optical methods^24–27^, our study observes this interaction directly in electronic transport measurements, shedding light on the ways excitons are involved in the conservation of momentum during tunnelling. Beyond its fundamental interest, our work establishes a platform for the investigation of the physical processes involved in the electrical generation of excitons in TMDs.

**Fig. 1:**
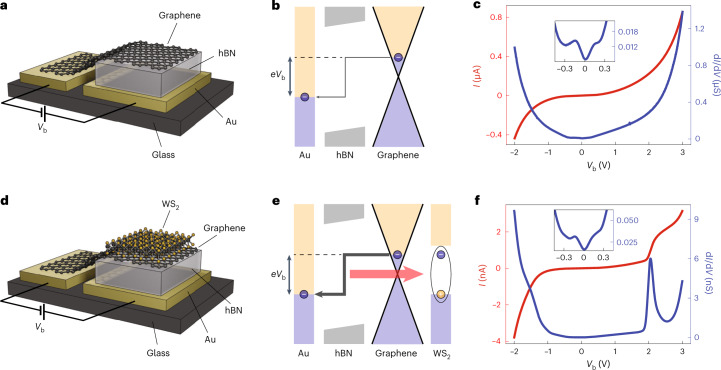
Device schematics, band diagrams and *I*−*V* characteristics. **a**, Illustration of a graphene/hBN/Au tunnelling device. The device is protected by a top hBN layer (not shown for better visibility). A bias voltage *V*_b_ applied between the graphene and gold electrodes gives rise to a tunnelling current through the hBN spacer. **b**, Band diagram of the device for positive *V*_b_. Electrons tunnel from graphene to Au both elastically (not shown) and inelastically (kinked arrow). **c**, Recorded *I*−*V* (red) and d*I*/d*V* (blue) curves at room temperature from the device in **a**. No distinctive features are observed besides an overall asymmetry due to the electronic density of states in Au. The inset shows a zoom into the low-bias region displaying phonon-assisted resonances. **d**, Illustration of a WS_2_/graphene/hBN/Au tunnelling device, which includes a protecting top hBN layer (not shown). **e**, Band diagram of the device at a positive bias. Electron tunnelling can be mediated by the creation of excitons (encircled electron–hole pair) in WS_2_, as indicated by the red arrow. **f**, Recorded *I*−*V* (red) and d*I*/d*V* (blue) curves at room temperature from the device in **d**. A new feature appears near *V*_b_ = 2.05 V. The inset shows a zoom into the low-bias region displaying phonon-assisted resonances.

Our reference device is illustrated in Fig. 1a, where the graphene and Au electrodes are separated by a 3–4 nm layer of insulating hBN. Applying a bias voltage between the two electrodes generates a tunnel current through the hBN barrier. The band diagram is depicted in Fig. 1b for a positive bias voltage *V*_b_. The inelastic electron tunnelling process, indicated by the kinked arrow, can be mediated by different modes of the structure, including phonons, defects, photons and surface plasmons^28–31^. The measured *I*−*V* curve of such a device is plotted in red in Fig. 1c, which features a nearly exponential dependence on *V*_b_ for both polarities, in agreement with previous reports^32,33^. To gain further insight, we evaluate the differential conductance d*I*/d*V*, shown in blue in Fig. 1c. This plot reveals an asymmetry in bias voltage (that is, the differential conductance increases more rapidly for negative *V*_b_). This can be understood by the abrupt increase of the electronic density of states in Au for negative bias voltages (Supplementary Fig. 9). In addition, some minute features can be observed near the zero bias region, as shown in the inset of Fig. 1c. The minimum appearing around *V*_b_ = 0 V is claimed to be a signature of inelastic electron tunnelling assisted by graphene phonons^14^. The latter mediate the in-plane momentum mismatch between the electronic states in Au and graphene^13–15,30^.

As shown in Fig. 1d, we place a TMD monolayer on top of the graphene electrode and investigate its influence on electron tunnelling. To avoid direct tunnelling between the TMD and Au, we place the TMD flake fully inside the graphene area. The band diagram of a WS_2_-based device is sketched in Fig. 1e for positive bias. Once *eV*_b_ (*e*, elementary charge) reaches the exciton energy of WS_2_, a new inelastic tunnelling channel opens up. It arises from the coupling of tunnelling electrons to WS_2_ excitons, as indicated by the red arrow. Figure 1f shows the measured *I*−*V* dependence (red curve) of the WS_2_ device. It exhibits a characteristic feature near *V*_b_ = 2 V, which is absent in the *I*−*V* curve of the reference device (Fig. 1c). We attribute this sudden increase in the tunnelling current to the onset of exciton-assisted resonant tunnelling. Note that this feature is not visible for negative *V*_b_ because it is masked by the high current arising from the large electronic density of states associated with 5*d* electrons in Au (Supplementary Fig. 9). For the same reason, the breakdown voltage of the device is considerably lower for negative *V*_b_ and we cannot extend our measurements beyond –2 V. Figure 1f shows the corresponding d*I*/d*V* curve (blue). For near-zero bias, it shows phonon-assisted resonances (inset), as in the case of the reference device. In contrast to the reference device, however, we now observe a pronounced resonance peak at ~2.05 V. The energy of 2.05 eV matches well with that of excitonic excitations in monolayer WS_2_ (refs. ^22,23^), which provides strong support in favour of the exciton-assisted tunnelling mechanism.

**Fig. 2:**
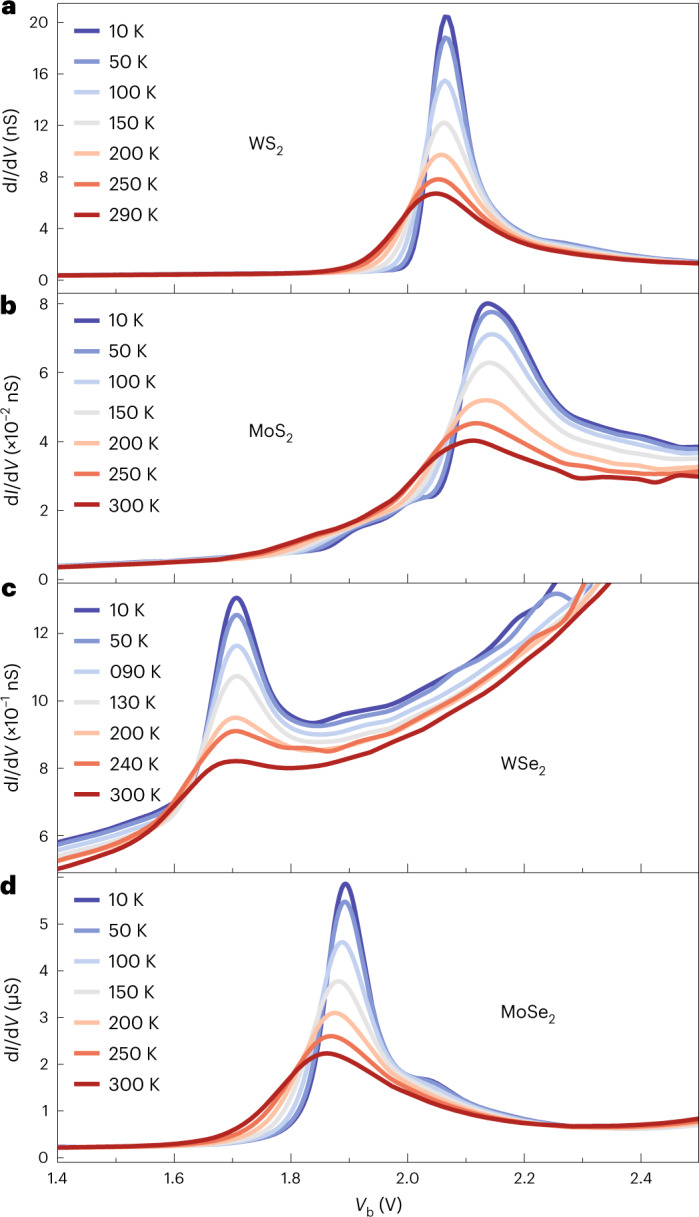
Temperature dependence of the tunnelling spectra. **a**–**d**, d*I*/d*V* measurements for WS_2_ (**a**), MoS_2_ (**b**), WSe_2_ (**c**) and MoSe_2_ (**d**) devices at temperatures indicated by the legends.

To substantiate our findings, we perform temperature-dependent measurements for devices with different types of TMD monolayers. Our results are shown in Fig. 2a–d for WS_2_, MoS_2_, WSe_2_ and MoSe_2_. For all of these TMDs, we observe that the resonance becomes sharper and stronger with decreasing temperature, an effect that we attribute to the lower thermal broadening of both the electron distribution in the electrodes^28^ and the exciton resonance.

For the WS_2_ device, we observe a shift in peak position to larger bias voltages, from ~2.05 V (290 K) to ~2.07 V (10 K), consistent with the temperature-dependent measurements of excitonic resonances in WS_2_ (refs. ^34,35^). Upon closer inspection, we find another resonance near 2.26 V, which becomes visible at low temperatures for the WS_2_ device (Fig. 2a and Supplementary Fig. 6a), suggesting coupling to excitons of higher energy.

Measurements for a MoS_2_ device are plotted in Fig. 2b, where a main peak at *V*_b_ = 2.1 V is observed, exhibiting a similar temperature dependence as the WS_2_ device. A weak feature appears at slightly lower *V*_b_ and develops into two distinct shoulders at lower temperatures, one at ~1.92 V and another one at ~2.02 V (also Supplementary Fig. 6b). The irregular features at bias voltages beyond 2.2 V, especially at high temperatures, can be attributed to measurement instabilities.

For the WSe_2_ device shown in Fig. 2c, we observe a main resonance at *V*_b_ = 1.7 V, and the temperature dependence is similar to the previous two devices. The wiggles appearing at higher bias voltages can be assigned to measurement instabilities (Supplementary Section 3 and Supplementary Figs. 6 and 7). The MoSe_2_ device plotted in Fig. 2d presents the same temperature behaviour. The resonance in this case appears at *V*_b_ = 1.9 V. The smaller feature at ~2.04 V, which becomes more pronounced at low temperatures, likely arises from higher energy excitons. The resonances appear at different bias voltages for every TMD, an observation that hints at exciton coupling.

**Fig. 3:**
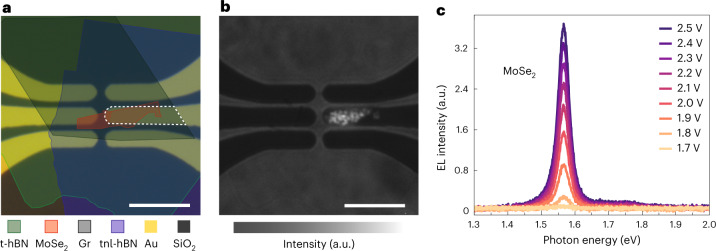
Radiative decay of tunnelling-induced excitons. **a**, Optical microscope image of a MoSe_2_ device fabricated on a glass (SiO_2_) substrate. The device consists of a vertical stack of a top hBN (t-hBN) protection layer, a MoSe_2_ monolayer, a graphene (Gr) monolayer, a ~3.3-nm-thick tunnel hBN layer (tnl-hBN) and a Au electrode. The graphene/hBN/Au tunnel junction is indicated by the white dashed line, where the graphene is partially covered by a MoSe_2_ monolayer (red polygon) on top. The upper two electrodes on the left serve as electrical contacts to the graphene sheet. Scale bar, 10 μm. **b**, Image of light emitted from the device at an applied voltage of 2.5 V, superimposed on the reference image of the device taken with back illumination. Scale bar, 10 μm. **c**, EL spectra for different bias voltages. The exciton peak at ~1.57 eV becomes more pronounced with increasing bias voltage.

Further evidence for exciton-assisted electron tunnelling is provided by our electroluminescence (EL) measurements. Excitons generated by resonant electron tunnelling can partially decay through radiative electron–hole recombination. As shown in Fig. 3c, this radiative decay gives rise to a distinctive peak in the measured EL spectrum. Figure 3a illustrates the layout of a MoSe_2_-based device. Electrons tunnel in the region where graphene, hBN and the Au electrode overlap (the area enclosed by a white dashed line). The MoSe_2_ monolayer indicated by the red polygon is placed directly above the graphene layer. The upper two electrodes on the left-hand side serve as electrical contacts to graphene. The photograph in Fig. 3b shows that EL is observed when a voltage *V*_b_ = 2.5 V is applied. The emission is restricted to the region where the MoSe_2_ monolayer overlaps with the tunnelling device. Spectra of the emitted light for different bias voltages are plotted in Fig. 3c. The peak centred at ~1.57 eV agrees with previous studies^36,37^ and can be assigned to the A exciton of MoSe_2_ according also to our photoluminescence spectroscopy measurements (Supplementary Table 2). Note, however, that the corresponding peak in the differential conductivity measurements shown in Fig. 2d appears at higher voltages than the A exciton energy, suggesting coupling to higher-order excitons. The EL intensity of the other three TMD devices in Fig. 2 is below our detection threshold because we used a considerably thicker hBN spacer to prevent breakdown, so the resulting current densities are more than two orders of magnitude lower than those of the MoSe_2_ device (Supplementary Fig. 1). Excitonic light emission from a WSe_2_ device with a thinner hBN tunnel barrier is shown in Supplementary Fig. 5.

**Fig. 4:**
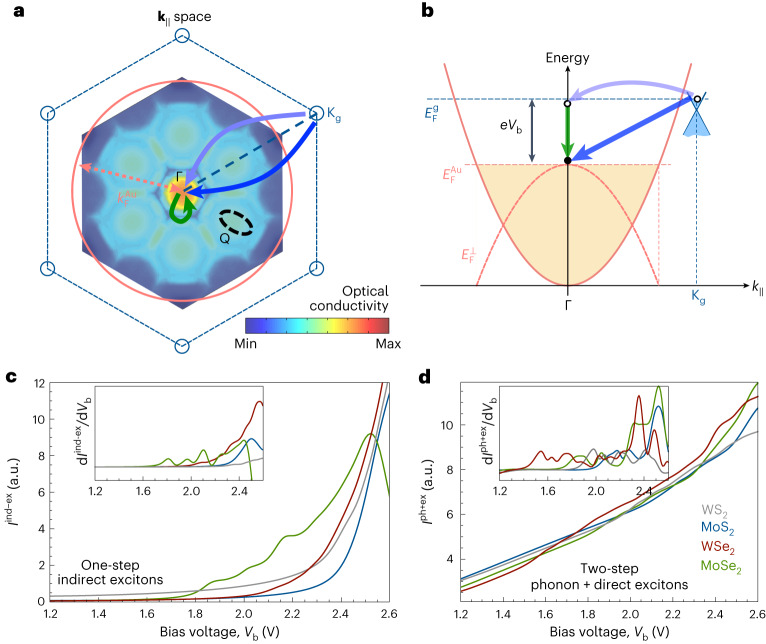
Exciton-assisted electron tunnelling pathways. **a**, We consider two possible tunnelling channels under the configuration of Fig. 1d, illustrated here by an on-scale representation of the graphene first Brillouin zone (1BZ; dashed hexagon in the space of parallel wave vector **k**_∥_), the surface-projected gold Fermi surface (orange circle, $k_{\mathrm{F}}^{\mathrm{Au}}$ is the Fermi wave vector of Au) and the TMD 1BZ (colour plot, showing the non-local surface conductivity for MoSe_2_ at 2.1 eV photon energy). The two channels are (1) one-step tunnelling assisted by the creation of indirect TMD excitons (blue arrow) and (2) two-step tunnelling associated with phonon creation (purple arrow) followed by direct-exciton creation (green arrow). Direct excitons produce the intense feature at the Γ point in the TMD conductivity, while indirect excitons show up as maxima at special regions of the TMD 1BZ, such as the Q point (colour plot, showing the non-local surface conductivity (logarithmic scale) for MoSe_2_ at 2.1 *eV* photon energy). **b**, Gold conduction-band dispersion diagram (orange), along with the energy–momentum region occupied by graphene electrons (light blue), involving a bias energy *eV*_b_ as well as the gold and graphene Fermi energies ${E}_{{{{\rm{F}}}}}^{{{{\rm{Au}}}}}$ and ${E}_{{{{\rm{F}}}}}^{{{{\rm{g}}}}}$, respectively, out-of-plane electron energy in gold at the Fermi level, $E_{\mathrm{F}}^{\perp}$. As a function of *k*_∥_, the out-of-plane gold Fermi surface (dashed orange parabola) has a minimum energy mismatch (that is, a maximum spill-out of gold electrons towards the hBN barrier) at the Γ point. The two considered tunnelling channels bridge the energy–momentum mismatch between the graphene K point and gold Fermi energy at *k*_∥_ = 0. The direction of ${\mathbf{k}}_{\parallel}$ in ${\bf b}$ is along the radial dashed line in ${\bf a}$. **c**,**d**, Calculated voltage-dependent tunnelling current associated with one-step indirect TMD exciton creation (**c**) and two-step phonon plus direct-exciton creation (**d**) for a hBN tunnel barrier of 3 nm and different TMDs. The insets in **c** and **d** show the corresponding d*I*/d*V* curves.

We continue by theoretically exploring the mechanisms responsible for resonant tunnelling and EL. The tunnelling process involves conservation of both energy and in-plane momentum, for which there is a clear mismatch between graphene and gold, as illustrated in Fig. 4a,b (also Supplementary Fig. 10). In addition, tunnelling favours final electron gold states with a large out-of-plane energy, which reside close to the conduction band of the hBN spacer. Consequently, the momentum of the final states lies near the surface-projected Γ point of the involved materials, as illustrated in Fig. 4b, and also in Supplementary Fig. 10c, where we observe a dramatic depletion of electron spill-out in gold-bound electrons when moving away from the Γ point, corresponding to **k**_∥_ = 0 in the space of parallel wave vector **k**_∥_. Tunnelling is therefore expected to be dominated by transitions from graphene electrons near the K_g_ point in this material to gold states near its Fermi level at the surface-projected Γ point, involving a large in-plane wave vector transfer of ~17 nm^−1^. Phonons can provide such large momentum, and in fact, graphene and hBN have similar in-plane lattice parameters (2% mismatch), so that phonons (frequency *ω*_p_) in both materials can assist quasi-elastic tunnelling, giving rise to the features observed at low bias voltages *V*_b_ ≈ *ħω*_p_/*e* ≈ 65 mV (*ħ*, reduced Planck’s constant) in the insets of Fig. 1c,f and in agreement with previous studies^14^.

We identify two tunnelling mechanisms that can bridge the graphene–gold momentum mismatch, as illustrated by thick arrows in Fig. 4a,b: (1) a single-step process (*I*^ind-ex^ current) involving the creation of indirect TMD excitons (blue arrow) and (2) a two-step process (*I*^ph+ex^ current) in which phonons provide the required large in-plane momentum (purple arrow) and direct excitons provide the energy difference between initial and final states (green arrow). A detailed analysis of these channels is provided in Supplementary Notes 6–9, from which we conclude that the associated currents bear a dependence on *V*_b_ given by

1a $${I}^{{{{\rm{ind}}}}-{{{\rm{ex}}}}}\propto \,{{{\rm{Im}}}}\left\{-{W}_{{{{\bf{G}}}}{{{\bf{G}}}}}({{{{\bf{k}}}}}_{\parallel },d,d,e{V}_{{{{\rm{b}}}}}/\hslash )\right\},$$

1b $${I}^{{{{\rm{ph}}}}+{{{\rm{ex}}}}}\propto \,{{{\rm{Im}}}}\left\{-{W}_{00}(0,d,d,e{V}_{{{{\rm{b}}}}}/\hslash -{\omega }_{{\mathrm{p}}})\right\},$$

expressed in terms of the screened interaction *W*_**GG**′_(**k**_∥_,*z*,*z*′,*ω*) (ref. ^38^). This screened interaction is defined as the potential created at a distance *z* from the gold surface by a unit charge placed at a distance *z*′, oscillating with frequency *ω*, and decomposed in parallel wave vectors **k**_∥_ within the first Brillouin zone (1BZ) of the TMD, as well as reciprocal lattice vectors **G** and **G**′. We set *z* = *z*′ = *d* at the graphene plane (therefore, *d* corresponds to the graphene-gold spacing), where the initial source of electrons is located. Also, the frequency *ω* is determined by the associated bias frequency *eV*_b_/*ħ* in *I*^ind-ex^, a value that needs to be corrected by the emitted phonon frequency in *I*^ph+ex^. The actual expressions that we use to compute the contributions in Fig. 4c,d are slightly more complex, as detailed in the Supplementary Information, but essentially captured by equations (1a) and (1b).

The single-step current *I*^ind-ex^ (equation (1a)) receives equal contributions from each of the six smallest non-vanishing reciprocal lattice vectors **G** of the TMD, involving a dominant **k**_∥_ value in the 1BZ where the optical conductivity reaches a maximum (colour plot in Fig. 4a). These features are associated with indirect excitons connecting the K point of the TMD with the Q point. Although the relative lattice orientations of TMD and graphene are undefined in our samples, we note that ∣**k**_∥_ + **G**∣ provides the required ~17 nm^−1^ in-plane wave vector, and in fact, even after averaging over lattice orientations, indirect excitons produce discernible shoulders in the *V*_b_-dependent profile of the resulting current (Fig. 4c), which are better visualized in the d*I*/d*V* curves (inset). The single-step indirect-exciton-assisted current thus displays features similar to experiment, although a determination of their detailed energies demands further computations exceeding our current resources. In the two-step tunnelling process (equation (1b)), the screened interaction is evaluated at **k**_∥_ = 0 and **G** = 0, and the resulting current displays direct-exciton features (Fig. 4d), but their strengths relative to the background are small compared to those of the indirect-exciton mechanism.

Since it is revealed that not only phonons but also indirect excitons between K-point valence-band states and Q-point conduction-band states can provide the missing in-plane momentum for tunnelling in our devices, we finalize our study by comparing the peak positions of our d*I*/d*V* curves with direct and indirect exciton energies. Supplementary Table 2 includes experimental and calculated values of exciton energies and in-plane momentum values for various TMDs. The Mo-based TMDs present peaks that are higher in energy than A excitons, and they fit well with the reported values of K–Q indirect excitons, suggesting that mostly indirect exciton transitions contribute to the observed resonant tunnelling behaviour. In contrast, K–Q indirect exciton energies in the W-based TMDs are closer to the A exciton energies, and both of them appear near their corresponding d*I*/d*V* peak energies, making the distinction between them more difficult. Although a one-step indirect exciton transition is likely to be more efficient, the EL emission from our MoSe_2_ devices suggests the involvement of direct excitons. Such radiative exciton decay can happen either from the phonon plus direct-exciton transitions or by phonon-assisted luminescence from indirect excitons, as reported in previous studies^25^.

In conclusion, we have demonstrated exciton-assisted resonant electron tunnelling in graphene/hBN/Au tunnel junctions with a TMD monolayer placed in proximity to the graphene layer. This process manifests as an abrupt increase in the tunnel current when the bias electron energy *eV*_b_ matches an exciton energy, resulting in a resonance peak in the d*I*/d*V* curve. An observed blueshift of the resonant peak with decreasing temperature is consistent with that of the corresponding excitons revealed by optical spectroscopy^34,35^. We find that the exciton states giving rise to the main resonance peaks in the d*I*/d*V* curves are different for the four studied TMD devices. We theoretically explain our measurements by electron tunnelling mechanisms that involve either indirect or direct excitons. Indirect excitons can supply both the energy and in-plane momentum required to tunnel from graphene to gold, while direct excitons require additional in-plane momentum supplied by phonons. Our findings are further substantiated by optical measurements, which reveal excitonic light emission driven by inelastic electron tunnelling. In our devices, the TMD layer is placed outside of the electron tunnelling path, which allows us to suppress exciton generation by direct charge injection, in contrast with previous studies of excitonic emission in tunnel junctions where exciton generation by charge injection cannot be excluded^39,40^. Finally, this device structure provides a platform not only for studying fundamental aspects of tunnelling processes, but also for exploring novel device functionalities for optoelectronics, all-electrical sensing and spectroscopy.

## Methods

### Sample fabrication

All flakes are mechanically exfoliated from bulk crystals in air (hBN) or in an argon-based glove box (TMD and graphene). The heterostructures are first assembled using a standard pick-up technique with a polydimethylsiloxane/polycarbonate stamp^41^, then transferred onto the prefabricated Au electrodes on a glass substrate in the glove box.

### Electrical and optical measurements

Room temperature electrical measurements are performed using a Keithley 2602B source meter. Low-temperature measurements are performed in a variable temperature probe station. Currents are measured using a Femto DDPCA-300 current amplifier. An ADwin Pro II data acquisition and control system is used to apply the bias voltage and read the output voltage of the current amplifier. For optical measurements, the samples are mounted on a Nikon TE300 inverted microscope under ambient conditions. The emitted light is collected by a ×100 objective (numerical aperture = 0.9) and analysed using an Andor iXon Ultra camera and a Princeton Instruments Acton SpectraPro 300i spectrometer.

### Theoretical calculations

The screened interaction in equation (1) is obtained by combining first-principles calculations of the non-local conductivity of TMD monolayers, the random-phase-approximation non-local response of graphene and the specular-reflection model for the non-local response of the gold surface based on the Lindhard permittivity for the bulk metal. The anisotropic response of hBN is accounted for through a local permittivity tensor. To deal with indirect TMD excitons, the response of the heterostructure is calculated with the inclusion of umklapp processes for the reflection and transmission coefficients of the TMD layer, which permeate the screened interaction, such that it becomes a tensor labelled by TMD reciprocal lattice vectors and wave vectors within the 1BZ. The derivation of equation (1) further involves the analysis of the electron potential landscape in the heterostructure, which is incorporated through the corresponding electron Green function. A detailed self-contained analysis of these elements is presented in Supplementary Notes 6–9, along with graphical information on the TMD non-local conductivities and the screened interaction of our heterostructures.

## Online content

Any methods, additional references, Nature Portfolio reporting summaries, source data, extended data, supplementary information, acknowledgements, peer review information; details of author contributions and competing interests; and statements of data and code availability are available at 10.1038/s41563-023-01556-7.

## Supplementary information

Supplementary Information Supplementary Figs. 1–19, Tables 1 and 2 and Discussion.

## Data Availability

All data presented in Figs. 1–4 are available via the ETH Zürich Research Collection at 10.3929/ethz-b-000607538.
