# Supplementary material for: Exciton-assisted electron tunnelling in van der Waals heterostructures
Source: Nat Mater. 2023 Jun 26;22(9):1094–9. doi: 10.1038/s41563-023-01556-7 (PMC10465355; doi:10.1038/s41563-023-01556-7)
Supplement: Supplementary file 1 — Supplementary Figs. 1–19, Tables 1 and 2 and Discussion. [file 41563_2023_1556_MOESM1_ESM.pdf]

nature materials

Article
https://doi.org/10.1038/s41563-023-01556-7

Exciton-assisted electron tunnelling in van der Waals heterostructures

In the format provided by the authors and unedited

Contents

S1. Device details and I–V characteristics 2

S2. Data for additional devices 3

S3. Discrimination between actual features and measurement instabilities 3

S4. Graphene/hBN/Au tunnel junction: comparison between theory and experiment 4

S5. Excitonic light emission from a WSe_2 device with a thinner hBN tunnel barrier 5

S6. Theory of inelastic electron tunneling between graphene and gold 5
- A. Preliminary considerations regarding energy–momentum conservation 5
- B. Vertical potential landscape, graphene electrons, and electron Green function 9
- C. Inelastic electron tunneling assisted by excitations in the heterostructure 10
- D. Indirect exciton-assisted one-step tunneling 11
- E. Two-step tunneling assisted by phonon and exciton creation 13
  - 1. Quasi-elastic phonon-assisted transitions 14
  - 2. Phonon+exciton-assisted tunneling 15

S7. Screened interaction under the in-plane isotropic and translational-invariant approximations 16

S8. Screened interaction including the atomic lattice periodicity 18

S9. First-principles nonlocal optical response of TMD monolayers 20

References 24

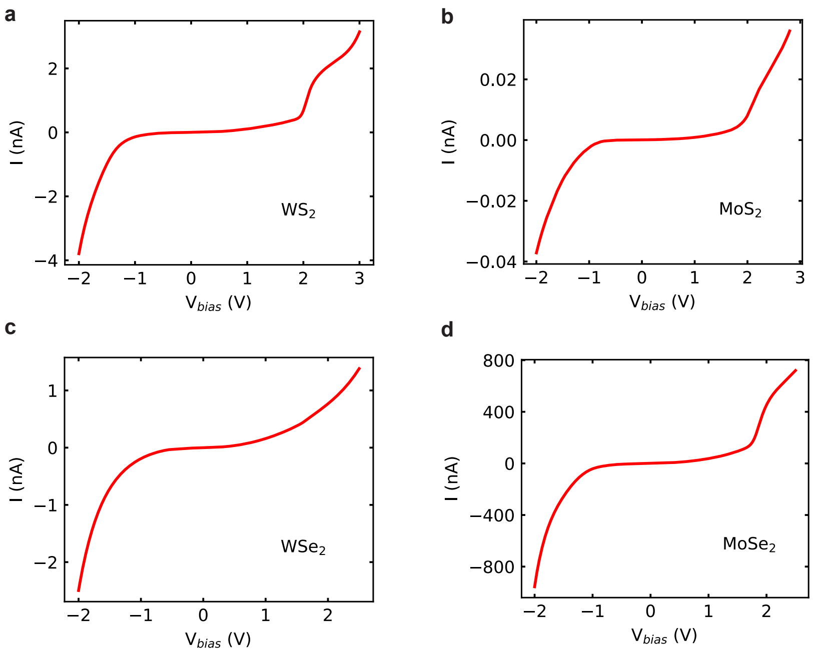

FIG. S1. Measured I–V characteristics of the four TMD devices discussed in the main text at room temperature.

S1. Device details and I–V characteristics

Geometrical details of the four devices discussed in the main text are provided in Table S1. The device size is determined by the overlap region of graphene, the hBN tunnel barrier, and the Au electrode. The TMD coverage is defined as the percentage of the device area under a TMD monolayer. The thickness of the hBN tunnel barrier is ∼3–4 nm, as estimated from the combination of the optical contrast and AFM measurements. However, the exact number of hBN atomic layers is difficult to determine with our AFM setup.

Device name	WS_2	MoS_2	WSe_2	$MoSe_2$
Device size (µm × µm)	$\sim 2.5 \times 5$	$\sim 2.5 \times 4$	$\sim 2.5 \times 10$	$\sim 2.5 \times 12$
TMD coverage (%)	~ 73	~ 70	~ 35	~ 50

TABLE S1. Sizes and TMD coverages of the devices.

The measured I–V characteristics of the devices are plotted in Fig. S1. The exciton-assisted tunneling feature is less visible in the WSe_2 device than in the other three devices due to a smaller TMD coverage. Nevertheless, the exciton signature is clearly observed in the dI/dV spectrum of this device, as shown in the main text. The current of the $MoSe_2$ device at a given bias voltage is more than two orders of magnitude larger than that of the other three devices due to a thinner hBN tunnel barrier. An exciton-assisted light emission can be measured for this device and is shown in the main text, while no light emission is detected for the other three devices. Assuming a similar electron-to-photon efficiency for all of our TMD devices, the number of emitted photons is at least two orders of magnitude smaller in the other three devices than in the $MoSe_2$ device at a given bias voltage, and thus, the electroluminescence signal lies below the detection limit of our setup.

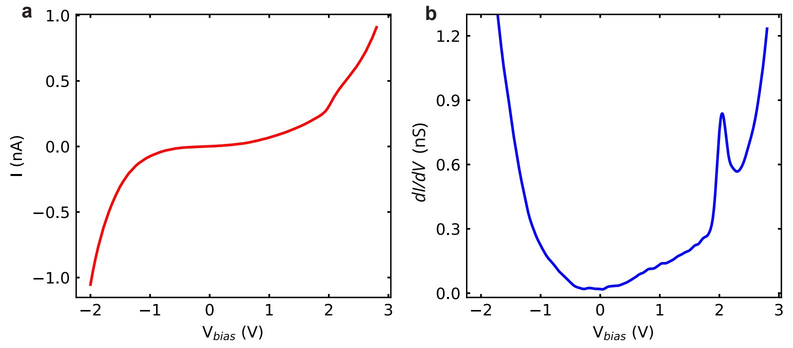

FIG. S2. Measured I–V characteristics (**a**) and corresponding dI/dV curve (**b**) of a second WS_2 device at room temperature.

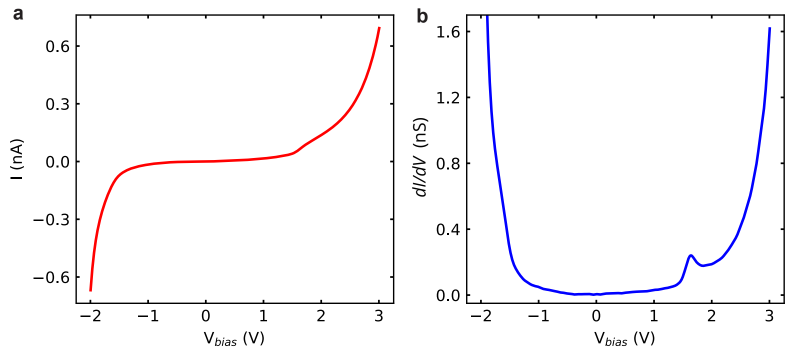

FIG. S3. Measured I–V characteristics (**a**) and corresponding dI/dV curve (**b**) of a second WSe_2 device at room temperature.

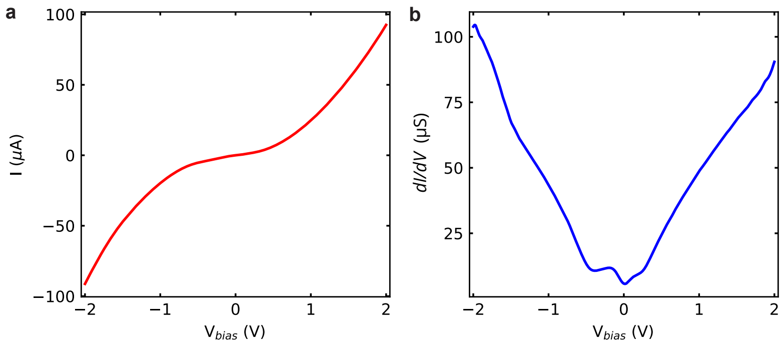

FIG. S4. Measured I–V characteristics (**a**) and corresponding dI/dV curve (**b**) of a WSe_2 device with a thinner ($\sim$ 2.3 nm) hBN tunnel barrier at room temperature.

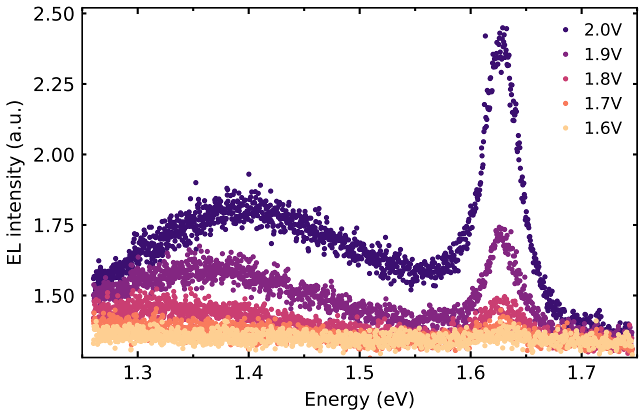

FIG. S5. Measured spectra of the light emitted by the WSe_2 device of Fig. S4 at different bias voltages.

S2. Data for additional devices

To further corroborate our findings, we fabricated two more devices, from which the results shown in the main text are reproduced. The I–V and dI/dV curves of a second WS_2 device and a second WSe_2 device are displayed in Figs. S2 and S3, respectively. Resonance features can be clearly observed in the dI/dV spectra, at $\sim$2.05 V for WS_2 and $\sim$1.64 V for WSe_2, which are essentially the same results as we find in the devices discussed in the main text. The reproducibility of the results further supports our exciton-assisted tunneling interpretation.

S3. Discrimination between actual features and measurement instabilities

In this section, we are presenting dI/dV curves for the four TMD devices (WS_2, MoS_2, WSe_2, and $MoSe_2$) measured at 10 K after (Fig. S6a-d) and before (Fig. S6e-h) filtering noise by means of a Savitzky–Golay filter. We conclude that the features that appear at high voltages in the dI/dV curve of the WSe_2 device in Fig. S6c can be attributed to measurement instabilities and increased noise. This becomes apparent in the unfiltered version of the measurements in Fig. S6g, where increased noise and fluctuations are observed. Finally, the features appearing close to the exciton resonances for the rest of the devices (WS_2, MoS_2, and $MoSe_2$) are still observed in the unfiltered data. To further support this interpretation, we show in Fig. S7a the MoS_2 dI/dV curve at both 50 K and 10 K, with features appearing consistently in both of them, suggesting that they are physical. This is in contrast to the dI/dV curve of the MoS_2 device in Fig. S7b, where the high-energy features vary for different measurements at 50 K and 10 K, suggesting that they are due to instabilities.

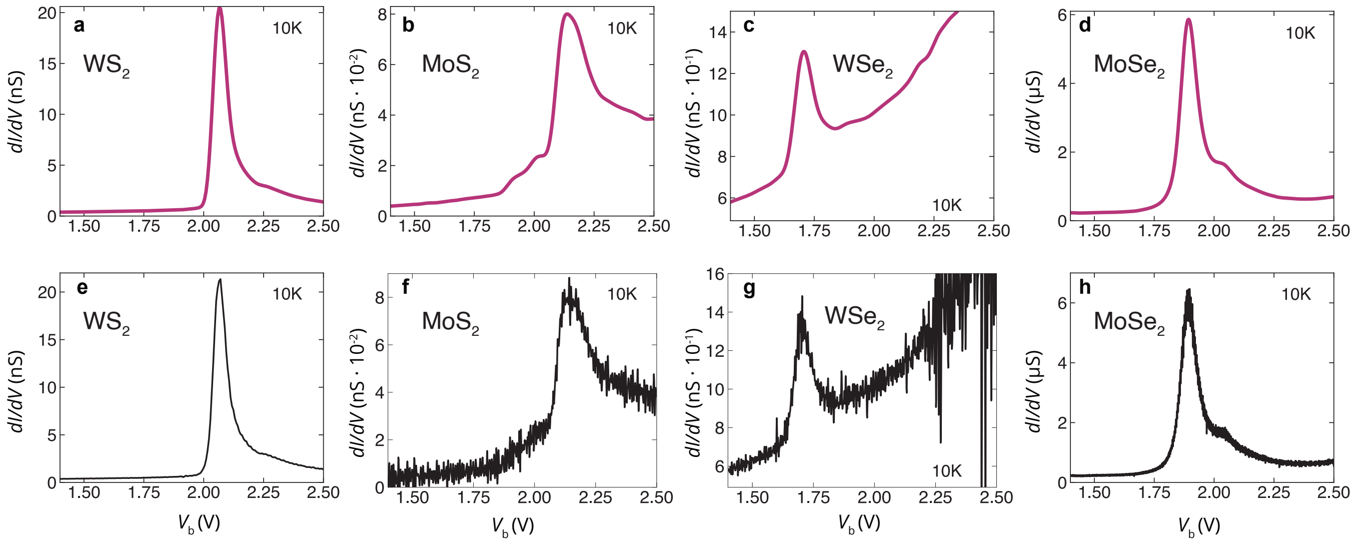

FIG. S6. **Measured dI/dV curves at 10 K for the various TMDs studied in this work.** Curves in panels (**a-d**) are treated with a Savitzky–Golay filter, whereas the unfiltered data are presented in (**e-h**). Features observed at V_b >2.2 V appear to be artificial due to the excess noise at those voltages, as shown in (**g**).

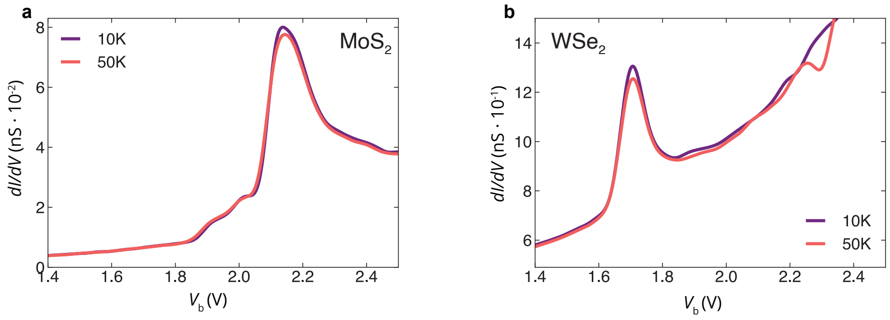

FIG. S7. **Comparison between 50 K and 10 K measurements for the dI/dV curves of (a) MoS$_2$ and (b) WSe$_2$ devices.** In (**a**), some features appear consistently in both measurements close to the exciton resonance, whereas in (**b**), features at higher energies fluctuate, suggesting that they could originate in measurement instabilities.

S4. Graphene/hBN/Au tunnel junction: comparison between theory and experiment

In the tunnel junctions presented in the main text, we explore a range of voltages up to 3 V. To further our understanding on whether we measure contributions from Fowler-Nordheim (F-N) tunneling or thermionic effects, we use a simple tunneling model with an infinitely high tunneling barrier to compare to our measurements. The phonon-induced tunneling current between graphene (Gr) and Au is calculated as [1]

$$I \propto \int_{\hbar\Omega}^{eV_b} \mathcal{T}^2 \cdot \rho_{\mathrm{Au}} \cdot \rho_{\mathrm{Gr}}(E)\, dE, \tag{S1}$$

where $\hbar\Omega$ is the phonon energy, $\mathcal{T}$ is the transmission matrix element (assumed to be constant in accordance with the DFT calculations presented in the supplementary material of Ref. [1]), ρ_{Au} is the DOS of Au (also assumed to be constant for $V_b > 0$ according to the DFT calculations in Fig. S9), and ρ_{Gr} is the DFT-calculated DOS of graphene taken from Ref. [2] and shifted in energy according to the level of graphene doping ($\sim$ 0.3 eV [1]). By taking the derivative of the calculated current as a function of voltage, we obtain the dI/dV curve shown in Fig. S8 (red dashed curve). We scale the calculation to fit the low-voltage region of the measured dI/dV curve. This is the region in which contributions of F-N or thermionic effects should be limited. Our comparison reveals that the calculated dI/dV curve closely follows our measurement up to 2 V, with significant deviations only above 2.5 V. These high-voltage contributions, which may be the result of F-N or thermionic effects, seem to become relevant at voltages outside the

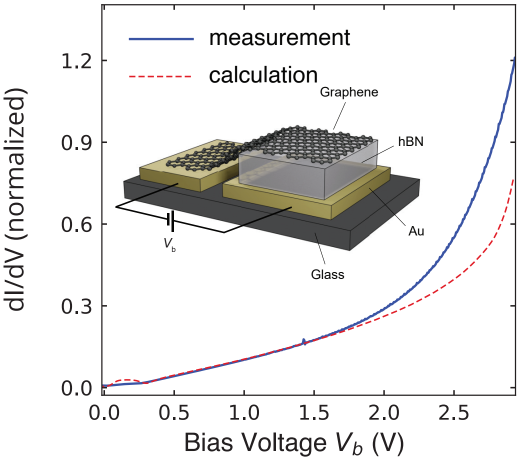

FIG. S8. **Comparison between dI/dV measurements and a simple tunneling model with an infinitely high tunneling barrier.** The model follows the measurement up to 2 V, with significant deviations only appearing above 2.5 V. The device structure under consideration is illustrated in the inset.

range in which we explore exciton energies (1.5 − 2.25 V).

S5. Excitonic light emission from a WSe_2 device with a thinner hBN tunnel barrier

Here, we investigate another WSe_2 device that has a thinner hBN tunnel barrier ($\sim$ 2.3 nm). The measured I–V profile is plotted in Fig. S4, showing a much larger tunnel current than in the devices with thicker tunnel barriers discussed above. In the dI/dV spectrum, the phonon-related feature shows up near zero bias voltage [3], but no clear exciton signatures can be observed. We attribute this result to the fact that the background arising from the larger total current overshadows any signature from the much smaller exciton-assisted tunneling current [4]. However, exciton-assisted light emission is observed from this device, as shown in Fig. S5. The peak at $\sim$1.63 eV in the electroluminescence spectra matches well the WSe_2 A exciton ground state [5], thus pointing to a coupling of the tunneling electrons to excitons in the WSe_2 monolayer. The bump feature around 1.4 eV in the spectra can be attributed to the broad light emission resulting from the coupling of tunneling electrons to optical modes such as surface-plasmon polaritons in the device [1].

S6. Theory of inelastic electron tunneling between graphene and gold

A. Preliminary considerations regarding energy–momentum conservation

We first note that the electronic density of states (DOS) of gold near the Fermi level is dominated by the 6sp-band, which is well-described by the free-electron gas (FEG) model up to energies $\sim$ 3 eV above the Fermi level (see Fig. S9). The contribution of the 5d band shows up as a sharp increase in the DOS below ~ -2 eV, which explains the sharp jump in the tunneling current observed in the experiment at negative biases exceeding that value. For the remainder of this discussion, we focus however on the smooth region dominated by the sp band, which we describe in the FEG model.

In a simplified picture, the surface-momentum-projected sp band of gold defines a circle of radius given by the Fermi wave vector $k_{\rm F}^{\rm Au}$, which we compare to the conduction band of graphene in Fig. S10a. This plot shows that graphene electrons near its Fermi level are situated far from the Γ point, where gold conduction electrons are expected to extend further beyond the metal surface. A band projection on energy and parallel momentum along the ΓK_g direction of n-type doped graphene (Fig. S10b) also illustrates how the electrons of this material need to bridge a large momentum mismatch to tunnel to gold states near the Γ point. This mismatch is smaller for transitions to gold states with high parallel wave vectors, but then, a huge jump in out-of-plane energy is encountered. In this respect, the landscape of the conduction band bottom (CBB) in the hexagonal boron nitride (hBN) layer [6] separating graphene from gold in our samples could play a role, whose analysis would require a realistic description of the electronic states in the system beyond the scope of the present theoretical model. However, a quantitative argument can be constructed upon

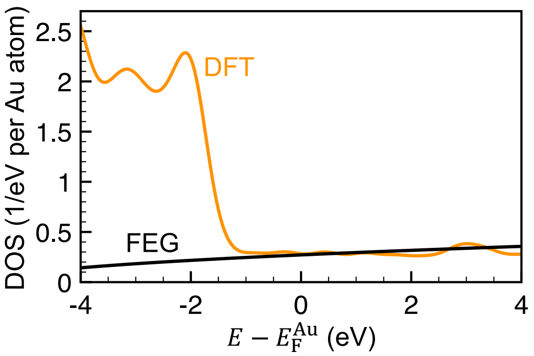

FIG. S9. **Density of states (DOS) of gold.** We compare density-functional-theory (DFT) calculations (including a Gaussian energy smearing of full width at half maximum $\sigma = 0.3$ eV) with the free-electron gas (FEG) model. In the latter, the DOS is $\rho_E = (m^*_{\rm Au}/\pi^2\hbar^3)\sqrt{2m^*_{\rm Au}E}$ and the Fermi energy is $E_F^{\rm Au} = (12\pi^2)^{2/3}(\hbar^2/2m^*_{\rm Au}a^2) \approx 5.52$ eV, where $a = 4.08$ Å is the lattice constant and $m^*_{\rm Au} \approx m_e$ is the effective mass.

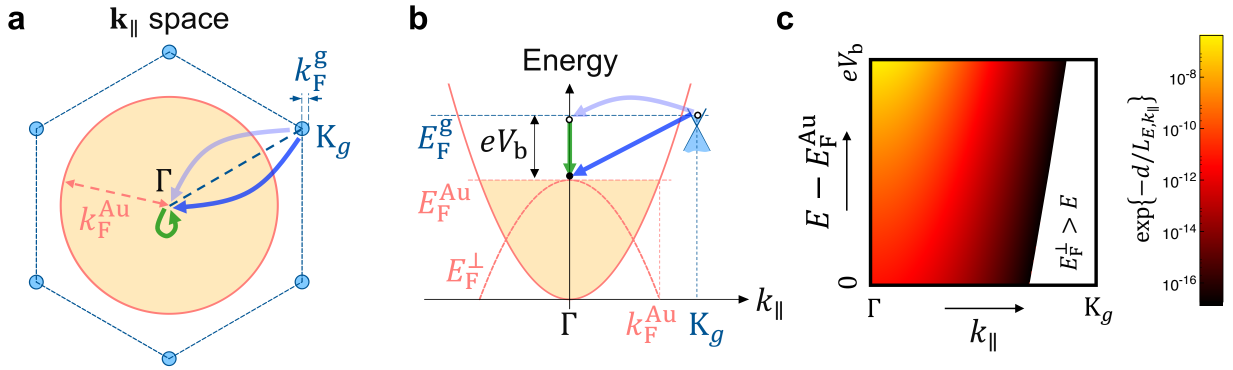

FIG. S10. **Electron tunneling pathways.** (**a**) On-scale representation of the in-plane electron momentum distribution in graphene (light blue) and gold (orange), including their respective Fermi wave vectors. Graphene is taken to be negatively doped to a Fermi energy of 0.5 eV relative to the Dirac points. (**b**) Energy–parallel-momentum diagram corresponding to the configuration in (**a**) for a graphene–gold bias energy $eV_b = 3$ eV. The downward dashed parabola shows the out-of-plane electron energy in gold at the Fermi level, $E_F^\perp = E_F^{\rm Au} - \hbar^2 k_\parallel^2/2m^*_{\rm Au}$, as a function of parallel wave vector $k_\parallel$. (**c**) Exponential attenuation of gold conduction electrons across the interface with an hBN film (thickness $d = 3$ nm) as a function of energy and in-plane wave vector, as determined by the spill-out distance $L_{E,k_\parallel} \approx \hbar\,[2m^*_{\rm hBN}(E_F^{\rm Au} + E_g/2 - E + \hbar^2 k_\parallel^2/2m^*_{\rm Au})]^{-1/2}$, estimated from a one-dimensional potential barrier determined by the hBN gap at the Γ point [6] $E_g \approx 8.9$ eV and the effective mass along the out-of-plane ΓA direction $m^*_{\rm hBN} \approx 0.63\,m_e$. In (**a**) and (**b**), we consider two possible channels for graphene-to-gold tunneling: (1) one-step direct inelastic transitions to near-threshold gold states around the surface projected Γ point (coinciding with the Γ point of graphene) assisted by the creation of indirect excitons in a neighboring TMD monolayer (dark blue arrows); and (2) two-step quasi-elastic tunneling from graphene to gold states near the surface-projected Γ point assisted by large-momentum phonon excitation (purple arrows), followed by quasi-vertical inelastic decay to states in the vicinity of the Fermi level of gold mediated by direct excitons (green arrows).

examination of the exponential attenuation factor displayed by the wave functions of gold conduction electrons across a distance of 3 nm outside the metal in the interface with hBN. The corresponding plot (Fig. S10c) clearly indicates that tunneling should be exponentially attenuated for energies and wave vectors deviating from the conditions of both quasi-elastic tunneling ($E - E_F^{\rm Au} \lesssim eV_b = 3$ eV in the figure) and electron momentum in the surface-projected gold Γ point.

Based on these arguments, we expect tunneling in the TMD/graphene/hBN/Au sandwich for positive bias of the graphene (as considered in the experiment) to involve a momentum transfer to graphene electrons such that they can transition to gold states near the Γ point (since graphene does not have any states near such point). The required wave vector transfer, $k_\parallel \sim \Gamma K_g = 4\pi/(3\sqrt{3}a_{\rm CC}) \approx 17.02$ nm^{-1}, dictated by the graphene lattice and the carbon-carbon bond distance $a_{\rm CC} \approx 0.1421$ nm, is too high to be supplied by the creation of low-momentum optical modes such as direct excitons. For example, the nonlocal conductivities of the four studied TMDs show a strong attenuation for $k_\parallel \gtrsim 0.5$ nm^{-1} (see Fig. S18 in Sec. S9). An additional source of momentum is thus required, for which we postulate

two possible channels that can assist graphene-to-gold tunneling:

- *Direct one-step transitions assisted by indirect TMD excitons.* Indirect excitons in the TMD monolayer can be formed by involving an electron and a hole of very different wave vectors. Upon examination of the possible indirect excitons connecting flat points in the band structure of TMD monolayers (i.e., those capable of producing Van Hove singularities), we find $\mathrm{KQ}_{\mathrm{TMD}}$ excitons (consisting of one hole at the K point and one electron at the Q point of the TMD) as good candidates to contribute to the observed tunneling current because their separation in wave vector is close to the $\Gamma\mathrm{K}_g$ distance in graphene (see Table S2). In addition, our calculated energies for $\mathrm{KQ}_{\mathrm{TMD}}$ excitons are in relatively good agreement with the observed peaks in our conductance measurements, as shown in Table S2, where we also include the corresponding $\mathrm{KQ}_{\mathrm{TMD}}$ gap energies (again not too far from the observed peaks), as well as information on the A and B excitons. For reference, we show the first Brillouin zone (1BZ) of $MoSe_2$ (pink) in the following scheme, superimposed on the 1BZ of graphene and the surface-projected gold Fermi surface taken from Fig. S10a:

  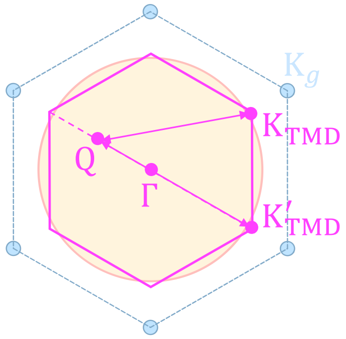

  In Table S2, we show values of the $\mathrm{KQ}_{\mathrm{TMD}}$ and $\mathrm{K}'\mathrm{Q}_{\mathrm{TMD}}$ distances, indicated by double arrows in this scheme. Although the relative azimuthal orientation between TMD and graphene lattices is not well defined in our samples, the set of $\mathrm{KQ}_{\mathrm{TMD}}$ distances spanned by the combinations of inequivalent K_g and Q points is dense, and some of them lie close to the required graphene $\Gamma\mathrm{K}_g$ momentum transfer, particularly considering that $\mathrm{KQ}_{\mathrm{TMD}}$ excitons span a wave vector distribution of finite extension in the $\sim 1\ \mathrm{nm}^{-1}$ range (see Fig. S19 in Sec. S9).

- *Two-step processes assisted by phonon excitation and direct TMD excitons.* Phonon excitation can bridge the graphene $\Gamma\mathrm{K}_g$ momentum mismatch, as both hBN and graphene have relatively close lattice parameters, and in addition, the electronic and phononic Brillouin zones are identical within each of them. The involved phonon energies are ~ 70 meV and $\sim 120 - 150$ meV [7, 8], much smaller than the exciton energies considered in this work. Apart from indirect excitons (see previous point), we cannot find a single source of momentum and energy that can simultaneously break the energy-momentum mismatch between graphene states and gold conduction electrons near the Fermi level at the Γ point. However, a two-step process can be invoked, involving a first quasi-elastic transition from graphene to gold near the Γ point, assisted by the excitation of a high-momentum phonon in graphene (or hBN, although proximity favors graphene phonons); and a subsequent inelastic decay from higher- to lower-energy states in gold (both of them near the Γ point), taking place by investing the transition energy in the creation of an exciton in the TMD monolayer placed on top of graphene. Inverting the order of these two steps would involve inelastic decay from graphene to an intermediate unoccupied gold state of large parallel momentum, and thus, this possibility should produce a negligible contribution because of the marginal spill out of such intermediate state near the Fermi level of gold (see Fig. S10c). For negative bias, with p-doped graphene, electrons could still tunnel from gold to graphene following a two-step process, but the intermediate and final states (both in graphene) have a small local DOS compared with gold, and thus, two-step inelastic tunneling should occur with lower probability in this direction.

We conclude these preliminary considerations by estimating the dependence on the graphene–gold bias voltage V_{b} of the shift in the graphene Fermi energy $E_{\mathrm{F}}^{\mathrm{g}}$ relative to the Dirac point energy E_{D} (i.e., relative to the graphene Fermi energy at zero bias). Throughout this work, we refer all energies to the CBB of gold, so we write the noted shift as $\Delta E_{\mathrm{F}}^{\mathrm{g}} = E_{\mathrm{F}}^{\mathrm{g}} - E_{\mathrm{D}}$. In addition, we assume hBN to fill the regions both below and above the TMD+graphene heterostructure, and ignore the effect of the TMD monolayer on graphene doping. A variational minimization of the total energy in the system shows that the graphene doping density n (electrons per unit area) is determined by the capacitor formed with gold as $n = (\epsilon_{\mathrm{hBN}}/4\pi e\, d)(V_{\mathrm{b}} - \Delta E_{\mathrm{F}}^{\mathrm{g}}/e)$, where $\epsilon_{\mathrm{hBN}} \approx 3.76$ is the out-of-plane DC permittivity of hBN, d is the hBN spacing layer thickness separating graphene from gold, and the effective bias voltage needs to be

material	A, B excitons (PL)	KQ gap (experiment)	conductance peak (our experiment)	direct excitons (experiment)	A, B excitons (our theory)	ΓK (nm^{-1})	ΓQ (nm^{-1})	KQ (nm^{-1})	K′Q (nm^{-1})
**WS_2**	1.98, -	2.04 [9]	2.07	2.00, 2.40 [10]<br>2.09, 2.23 [11]<br>2.06, 2.20 [12]<br>2.08 [13]	1.92, 2.35	13.13	6.94	17.65	20.07
**MoS_2**	1.87, 2.0	2.41 [14]	2.10	1.87, 2.03 [10]<br>1.92, 2.08 [15]<br>1.94, 2.11 [12]	2.05, 2.19	13.13	6.46	17.28	19.58
**WSe_2**	1.64,-	1.69 [16]<br>2.09 [14]	1.70	1.65, 2.05 [10]<br>1.65, 2.08 [5]<br>1.72, 1.85 [17]	1.50, 1.97	12.48	6.94	17.05	19.43
**$MoSe_2$**	1.57, 1.72	1.84 [18]<br>2.34 [14]	1.90	1.55, 1.75 [10]<br>1.64, 1.79 [19]<br>1.64, 1.79 [20]<br>1.64, 1.81 [12]	1.80, 2.00	12.62	6.60	16.92	19.23

TABLE S2. **Direct and indirect excitons in TMD monolayers.** We present a compilation of energies for the measured KQ_{TMD} gaps (from photoluminescence [9, 16, 18] and I–V [14] studies), the conductance peaks in our experiment, luminescence measurements from different sources, and our calculated A and B excitons. We show data for the four different TMD monolayer materials under consideration. All energies are in eV. We also provide wave vector distances between singular points of the 1BZ (four rightmost columns). The Q point is defined by an intermediate conduction band minimum along the ΓK_{TMD} line. We consider two of the four distances between K and Q points in the 1BZ of each TMD, whose values lie close to the ΓK_g distance in graphene (17.02 nm^{-1}).

reduced by $\Delta E_F^g/e$ to account for the change in energy needed to add/subtract every new electron to/from graphene as its Fermi energy is varied. Using the additional relation [21] $\Delta E_F^g = \hbar v_F^g \sqrt{\pi n}$ for positive bias, where $v_F^g \approx 10^6$ m/s is the graphene Fermi velocity, we finally obtain the Fermi energy shift as

$$\Delta E_F^g = \frac{E_0}{2}\left(\sqrt{1+\frac{4eV_b}{E_0}}-1\right) \tag{S2}$$

with $E_0 = \epsilon_{hBN}(\hbar v_F^g/2e)^2/d$. From this expression, we have, for example, $\Delta E_F^g \approx 0.49$ eV for $d = 3$ nm and $eV_b = 3$ eV ($\gg E_0 \approx 0.09$ eV). Incidentally, this value is slightly shifted with respect to the large-bias limit $\Delta E_F^g \approx \sqrt{eV_b E_0} \approx$ 0.53 eV.

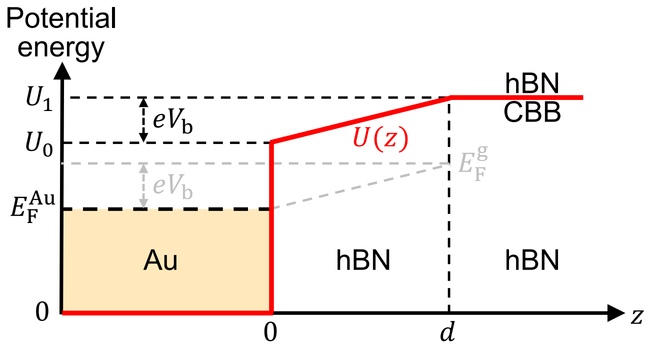

FIG. S11. **Electron potential landscape.** We model the out-of-plane electron dynamics through a one-dimensional potential energy $U(z)$, here sketched for a graphene–gold positive bias potential energy eV_b and defined by the conduction band bottom (CBB) of gold at $z < 0$, as well as the potential energies U_0 and U_1 of the CBB of hBN at $z = 0$ and $z = d$ (the graphene–gold spacing), respectively. The graphene Fermi level E_F^g is separated from the gold Fermi level E_F^{Au} by the bias potential energy $E_F^g - E_F^{Au} = eV_b$. We set $U_0 - E_F^{Au} \approx 4.45$ eV as half of the hBN gap at the Γ point [6].

B. Vertical potential landscape, graphene electrons, and electron Green function

We assume the tunneling electron to evolve in the potential energy landscape sketched in Fig. S11, as described by the Hamiltonian

$$\mathcal{H}_0 = -\frac{\hbar^2\nabla^2}{2m_{\rm e}} + U(z),$$

where a uniform isotropic electron mass is assumed for simplicity. We note that the strong variation of the electron mass and energy gap within the in-plane 1BZ of hBN [6] can produce substantial corrections to this model, which deserve a future investigation including a more detailed description of the electron states involved in the tunneling process. In addition, we approximate the wave function of each initial graphene electron i as a separable state

$$\varphi_i(\mathbf{r}) = \varphi_i(z)\mathrm{e}^{\mathrm{i}(\mathbf{K}_g+\mathbf{Q}_i)\cdot\mathbf{R}}/\sqrt{A}$$

of energy $\hbar\varepsilon_i = E_{\rm D} + s_i\hbar v_{\rm F}^{\rm g}Q_i$ [with the Dirac points at an energy $E_{\rm D} = E_{\rm F}^{\rm g} - \Delta E_{\rm F}^{\rm g}$, see Eq. (S2)] relative to the CBB of gold and wave vector $\mathbf{Q}_i$ relative to one of the K_g points of momentum $\mathbf{K}_g$ (see Figs. S10 and S11). Here, $s_i = \pm1$ refers to the upper (+) and lower (−) graphene Dirac cones, $\mathbf{K}_g$ and $\mathbf{Q}_i$ and both 2D vectors, the in-plane position coordinates are $\mathbf{R} = (x, y)$, and we introduce the graphene area A for normalization. We also note that $E_{\rm F}^{\rm g}$ is referred to the CBB of gold. Incidentally, the two inequivalent Dirac cones within the 1BZ should contribute equally to the tunneling current, and therefore, we absorb valley and spin degeneracies in an overall factor of 4.

The evolution of the tunneling electron in the potential landscape of Fig. S11 can be conveniently described in terms of the three-dimensional (3D) electron Green function $G_0(\mathbf{r},\mathbf{r}',\varepsilon)$ defined by $(\mathcal{H}_0 - \hbar\varepsilon)G_0(\mathbf{r},\mathbf{r}',\varepsilon) = -\delta(\mathbf{r}-\mathbf{r}')$. Due to the in-plane translational symmetry of the system, the Green function depends on $\mathbf{R}$ and $\mathbf{R}'$ only through the difference $\mathbf{R}-\mathbf{R}'$, so it can be written as

$$G_0(\mathbf{r},\mathbf{r}',\varepsilon) = \int \frac{d^2\mathbf{Q}}{(2\pi)^2}\,\mathrm{e}^{\mathrm{i}\mathbf{Q}\cdot(\mathbf{R}-\mathbf{R}')}\,G_0(z,z',\varepsilon-\hbar Q^2/2m_{\rm e}) \tag{S3}$$

in terms of the one-dimensional Green function $G_0(z,z',\varepsilon)$ satisfying $(\mathcal{H}_0 - \hbar\varepsilon)G_0(z,z',\varepsilon) = -\delta(z-z')$, which we correct in Eq. (S3) by subtracting the in-plane energy $\hbar^2Q^2/2m_{\rm e}$ from $\hbar\varepsilon$.

In the calculations presented below, we only need the Green function evaluated at $G_0(0,z,\varepsilon)$ and $G_0(z,d,\varepsilon)$, as well as the derivative of the former with respect to the first argument. It is then useful to point out that the spatial arguments of these functions are interchangeable in virtue of the reciprocity property $G_0(z,z',\varepsilon) = G_0(z',z,\varepsilon)$. In addition, we note that the required functions are solutions of $(\mathcal{H}_0 - \hbar\varepsilon)G_0(z,z',\varepsilon) = -\delta(z-z')$ for $z'=0$ and $z'=d$. This allows us to express them in terms of analytical functions within each of the three distinct regions of the potential $U(z)$ in Fig. S11:

$$G_0(z,z',\varepsilon) = \begin{cases} A(z')\,\mathrm{e}^{-\mathrm{i}kz}, & z<0, \\ B(z')\,\psi_1(z) + C(z')\,\psi_2(z), & 0\le z\le d, \\ D(z')\,\mathrm{e}^{-\kappa(z-d)}, & d<z, \end{cases} \tag{S4}$$

where we consider outgoing waves with $k = \sqrt{2m_{\rm e}\varepsilon/\hbar}$ and $\kappa = \sqrt{2m_{\rm e}(U_1/\hbar-\varepsilon)/\hbar}$, which are suitable solutions decaying at $|z|\to\infty$ for a source placed at $z'\in[0,d]$. The functions in the intermediate region can be written as

$$\psi_1(z) = \mathrm{Ai}(\theta),$$
$$\psi_2(z) = \mathrm{Bi}(\theta)$$

in terms of the Airy functions Ai and Bi [22] with an argument $\theta = (2m_{\rm e}eV_{\rm b}d^2/\hbar^2)^{1/3}\left[z/d + (U_0-\hbar\varepsilon)/eV_{\rm b}\right]$. Finally, the coefficients in Eq. (S4) are obtained from the conditions

$$A - B\psi_1(0) - C\psi_2(0) = 0, \tag{S5a}$$
$$D - B\psi_1(d) - C\psi_2(d) = 0, \tag{S5b}$$
$$\mathrm{i}kA + B\psi_1'(0) + C\psi_2'(0) = n_0\times 2m_{\rm e}/\hbar^2, \tag{S5c}$$
$$\kappa D + B\psi_1'(d) + C\psi_2'(d) = n_d\times 2m_{\rm e}/\hbar^2. \tag{S5d}$$

In particular, Eqs. (S5a) and (S5b) guarantee continuity at $z=0$ and $z=d$, respectively, whereas Eqs. (S5c) and (S5d) relate to the jump in the derivative $(2m_e/\hbar^2)$ produced by the inhomogeneous term $-\delta(z-z')$. More precisely, we need to set $n_0=1$ and $n_d=0$ for $z'=0$; and $n_0=0$ and $n_d=-1$ for $z'=d$. The required Green function values are then given by Eq. (S4) with coefficients A, B, C, and D determined by solving the 4×4 system of equations (S5).

Likewise, we evaluate $\partial_{z'}G_0(z',z,\varepsilon)|_{z'=0^-}$ by writing a solution similar to Eq. (S4),

$$\partial_{z'}G_0(z',z,\varepsilon)|_{z'=0} = \begin{cases} A'\,\mathrm{e}^{-\mathrm{i}kz}, & z<0,\\ B'\,\psi_1(z)+C'\,\psi_2(z), & 0\le z\le d,\\ D'\,\mathrm{e}^{-\kappa(z-d)}, & d<z,\end{cases}$$

with coefficients determined by the equations

$$A'-B'\psi_1(0)-C'\psi_2(0)=2m_e/\hbar^2, \tag{S6a}$$
$$D'-B'\psi_1(d)-C'\psi_2(d)=0, \tag{S6b}$$
$$\mathrm{i}kA'+B'\psi_1'(0)+C'\psi_2'(0)=0, \tag{S6c}$$
$$\kappa D'+B'\psi_1'(d)+C'\psi_2'(d)=0. \tag{S6d}$$

This solution if obtained by first writing $G_0(z',z,\varepsilon)$ (with $z'<0$ regarded as a parameter) in terms of z-dependent outgoing waves within the $z<z'$ and $z>d$ regions, as well as Airy functions for $0\le z\le d$ and $\mathrm{e}^{\pm\mathrm{i}kz}$ waves for $z'\le z<0$. We then take the derivative of the resulting coefficients with respect to z' and calculate the $z'\to0^-$ limit (i.e., approaching $z'=0$ from the left), which leads to Eqs. (S6).

C. Inelastic electron tunneling assisted by excitations in the heterostructure

The tunneling process can be assisted by the creation of an excitation in the heterostructure, which absorbs the energy and momentum change undergone by the transferred electron. In this section, we derive a general expression for the inelastic tunneling current that encapsulates such excitations n of energies $\hbar\omega_n$ in the dielectric response function. Although we consider electron tunneling from graphene to gold in this work, the formalism presented in this section can be applied to any combination of two conductive materials separated by an insulating layer and subject to a relative bias. Starting from an electron in an initial graphene state i of wave function $\varphi_i(\mathbf{r})$ and energy $\hbar\varepsilon_i$, the final electron wave function component of energy $\hbar(\varepsilon_i-\omega_n)$ associated with an additional excitation n is given by

$$\psi_{n,i}(\mathbf{r})=\int d^3\mathbf{r}'\;G_0(\mathbf{r},\mathbf{r}',\varepsilon_i-\omega_n)\,\langle n|\,\mathcal{H}_1(\mathbf{r}')\,|0\rangle\,\varphi_i(\mathbf{r}') \tag{S7}$$

within first-order perturbation in the interaction Hamiltonian $\mathcal{H}_1$ of matrix elements

$$\langle n|\,\mathcal{H}_1(\mathbf{r}')\,|0\rangle=-e\int d^3\mathbf{r}'\,\frac{\rho_{n0}(\mathbf{r}')}{|\mathbf{r}-\mathbf{r}'|}, \tag{S8}$$

sandwiched by the ground and excited states of the heterostructure, $|0\rangle$ and $|n\rangle$, respectively. Here, $\rho_{n0}(\mathbf{r})=\langle n|\,\hat\rho(\mathbf{r})\,|0\rangle$ is the matrix element of the charge density operator $\hat\rho(\mathbf{r})=\sum_j q_j\,\delta(\mathbf{r}-\mathbf{r}_j)$, incorporating all electrons and nuclei of charges q_j at positions $\mathbf{r}_j$ in the system. The Hamiltonian $\mathcal{H}_1$ thus describes the Coulomb interaction between such charges and the tunneling electron in a completely general fashion. The details of the structure are additionally captured by the unperturbed Green function $G_0(\mathbf{r},\mathbf{r}',\varepsilon)$, which remains general in this section, but we then specify it for the heterostructure under consideration by using the methods described in Sec. S6 B.

From the wave functions given by Eq. (S7), we obtain the tunneling current density (charge moving downwards and entering the gold surface per unit time and unit area) as

$$J=\frac{e\hbar}{m_e}\sum_{n,i}f_T(\hbar\varepsilon_i)\,\{1-f_T[\hbar(\varepsilon_i-\omega_n)]\}\,\mathrm{Im}\{\psi^*_{n,i}(\mathbf{r})\partial_z\psi_{n,i}(\mathbf{r})\}\big|_{z=0^-}, \tag{S9}$$

where we sum over all initial occupied states i and material excitations n that lead to final unoccupied gold states of energy $\hbar(\varepsilon_i-\omega_n)$, as imposed by using the Fermi–Dirac distribution f_T at temperature T. The gold surface is placed

at $z=0$, so we evaluate the current right below that plane at $z=0^-$ in Eq. (S9). From Eqs. (S7)–(S9), we find

$$J = \frac{e^3\hbar}{m_e}\sum_{n,i} f_T(\hbar\varepsilon_i)\left\{1-f_T[\hbar(\varepsilon_i-\omega_n)]\right\}\int d^3\mathbf{r}'\int d^3\mathbf{r}''\int d^3\mathbf{r}_1\int d^3\mathbf{r}_2 \tag{S10}$$
$$\mathrm{Im}\Big\{G_0^*(\mathbf{r},\mathbf{r}',\varepsilon_i-\omega_n)\,\partial_z G_0(\mathbf{r},\mathbf{r}'',\varepsilon_i-\omega_n)\big|_{z=0^-}\;\varphi_i^*(\mathbf{r}')\varphi_i(\mathbf{r}'')\,\frac{\rho_{0n}(\mathbf{r}_1)\rho_{n0}(\mathbf{r}_2)}{|\mathbf{r}'-\mathbf{r}_1||\mathbf{r}''-\mathbf{r}_2|}\Big\}.$$

Following a method similar to the derivation of the electron energy-loss probability in electron microscopy (see Ref. [23]), this expression can be written in terms of a response function by exploiting the identities [24]

$$\mathrm{Im}\{\chi(\mathbf{r},\mathbf{r}',\omega)\} = -\frac{\pi}{\hbar}\sum_n \rho_{0n}(\mathbf{r})\rho_{n0}(\mathbf{r}')\,\delta(\omega_n-\omega), \tag{S11a}$$

$$W^{\rm ind}(\mathbf{r},\mathbf{r}',\omega) = \int d^3\mathbf{r}_1\int d^3\mathbf{r}_2\,\frac{1}{|\mathbf{r}-\mathbf{r}_1|}\,\chi(\mathbf{r}_1,\mathbf{r}_2,\omega)\,\frac{1}{|\mathbf{r}'-\mathbf{r}_2|}, \tag{S11b}$$

where $\chi(\mathbf{r},\mathbf{r}',\omega)$ is the nonlocal susceptibility and $W^{\rm ind}(\mathbf{r},\mathbf{r}',\omega)$ is the induced part of the screened interaction. The latter is defined as the potential created at a position $\mathbf{r}$ by a time-dependent unit charge of magnitude $\mathrm{e}^{-\mathrm{i}\omega t}$ placed at $\mathbf{r}'$ (see Secs. S7 and S8 for explicit calculations of this quantity in the planar heterostructures under consideration). Using Eqs. (S11) to manipulate Eq. (S10), we obtain

$$J = \frac{e^3\hbar^2}{\pi m_e}\sum_i\int_0^\infty d\omega\; f_T(\hbar\varepsilon_i)\left\{1-f_T[\hbar(\varepsilon_i-\omega)]\right\}\int d^3\mathbf{r}'\int d^3\mathbf{r}''\;\mathrm{Im}\big\{-W(\mathbf{r}',\mathbf{r}'',\omega)\big\} \tag{S12}$$
$$\times\,\mathrm{Im}\Big\{G_0^*(\mathbf{r},\mathbf{r}',\varepsilon_i-\omega)\,\partial_z G_0(\mathbf{r},\mathbf{r}'',\varepsilon_i-\omega)\big|_{z=0^-}\;\varphi_i^*(\mathbf{r}')\varphi_i(\mathbf{r}'')\Big\},$$

where we have substituted the induced part of the screened interaction by the full interaction because $W(\mathbf{r},\mathbf{r}',\omega)-W^{\rm ind}(\mathbf{r},\mathbf{r}',\omega)=1/|\mathbf{r}-\mathbf{r}'|$ is a real function.

D. Indirect exciton-assisted one-step tunneling

We now apply Eq. (S12) to discuss one-step transitions from graphene states i assisted by the creation of indirect excitons in the TMD layer. In what follows, the sum over i is understood to be restricted to occupied graphene states and we set $T=0$, such that $1-f_T[\hbar(\varepsilon_i-\omega)]=\Theta[\hbar(\varepsilon_i-\omega)-E_{\rm F}^{\rm Au}]$ in expressed in terms of the step function Θ and the gold Fermi energy $E_{\rm F}^{\rm Au}$. Accordingly, we rewrite Eq. (S12) as

$$J^{\rm ind-ex} = \frac{e^3\hbar^2}{\pi m_e}\sum_i\int_0^\infty d\omega\;\Theta\big[\hbar(\varepsilon_i-\omega)-E_{\rm F}^{\rm Au}\big]\int d^3\mathbf{r}'\int d^3\mathbf{r}''\;\mathrm{Im}\big\{-W(\mathbf{r}',\mathbf{r}'',\omega)\big\} \tag{S13}$$
$$\times\,\mathrm{Im}\Big\{G_0^*(\mathbf{r},\mathbf{r}',\varepsilon_i-\omega)\,\partial_z G_0(\mathbf{r},\mathbf{r}'',\varepsilon_i-\omega)\big|_{z=0^-}\;\varphi_i^*(\mathbf{r}')\varphi_i(\mathbf{r}'')\Big\}.$$

Momentum mismatch is then considered to be bridged by large wave vectors $\mathbf{k}_\parallel$ in the screened interaction, contributed by indirect excitons in the TMD layer.

Assuming isotropy and translational invariance in the in-plane response, one can write the screened interaction as

$$W(\mathbf{r},\mathbf{r}',\omega) = \int\frac{d^2\mathbf{k}_\parallel}{(2\pi)^2}\,\mathrm{e}^{\mathrm{i}\mathbf{k}_\parallel\cdot(\mathbf{R}-\mathbf{R}')}\,W(k_\parallel,z,z',\omega) \tag{S14}$$

in terms of momentum components $W(k_\parallel,z,z',\omega)$. However, for the large values of $\mathbf{k}_\parallel$ under consideration, the in-plane atomic lattice can play a substantial role, imprinting a lattice periodicity on the screened interaction, which can be written as

$$W(\mathbf{r},\mathbf{r}',\omega) = \sum_{\mathbf{G}\mathbf{G}'}\int_{\rm 1BZ}\frac{d^2\mathbf{k}_\parallel}{(2\pi)^2}\,\mathrm{e}^{\mathrm{i}(\mathbf{k}_\parallel+\mathbf{G})\cdot\mathbf{R}}\mathrm{e}^{-\mathrm{i}(\mathbf{k}_\parallel+\mathbf{G}')\cdot\mathbf{R}'}\,W_{\mathbf{G}\mathbf{G}'}(\mathbf{k}_\parallel,z,z',\omega). \tag{S15}$$

This expression, which generalizes Eq. (S14) by considering $W_{\mathbf{G},\mathbf{G}'}(\mathbf{k}_\parallel,z,z',\omega)$ components labeled by TMD reciprocal lattice vectors $\mathbf{G}$ and $\mathbf{G}'$ and restricting the $\mathbf{k}_\parallel$ integral to the 1BZ, is general to describe the linear optical response

of a TMD layer including atomic periodicity. In our analysis, we neglect lattice contributions to the Green function as a less relevant effect than in the excitonic optical response of the TMD material.

Upon insertion of Eqs. (S3) and (S15) into Eq. (S13), noticing the plane-wave dependence of the wave functions on in-plane coordinates, we can readily carry out the integrals over $\mathbf{R}'$ and $\mathbf{R}''$. Further averaging the current over in-plane positions $\mathbf{R}$, we obtain

$$\begin{aligned} J^{\rm ind-ex} =& \frac{1}{A}\frac{e^3\hbar^2}{\pi m_{\rm e}}\sum_i\sum_{\mathbf{G}}\int_0^\infty d\omega\; \Theta\big[\hbar(\varepsilon_i-\omega)-E_{\rm F}^{\rm Au}\big] \\ &\times \int_{\rm 1BZ}\frac{d^2\mathbf{k}_\parallel}{(2\pi)^2}\int dz'\int dz''\;{\rm Im}\big\{-W_{\mathbf{G}\mathbf{G}}(\mathbf{k}_\parallel,z',z'',\omega)\big\} \\ &\times {\rm Im}\Big\{G_0^*(0,z',\varepsilon_i-\omega-\hbar|\mathbf{Q}_i-\mathbf{k}_\parallel+\mathbf{G}-\mathbf{K}_g|^2/2m_{\rm e}) \\ &\quad\times \partial_z G_0(z,z'',\varepsilon_i-\omega-\hbar|\mathbf{Q}_i-\mathbf{k}_\parallel+\mathbf{G}-\mathbf{K}_g|^2/2m_{\rm e})\big|_{z=0^-}\;\varphi_i^*(z')\varphi_i(z'')\Big\}, \end{aligned} \tag{S16}$$

where $\mathbf{K}_g$ is the wave vector at the graphene K_g point and we find contributions from different Brillouin zones labeled by $\mathbf{G}$. We note that the average over $\mathbf{R}$ has eliminated nondiagonal $W_{\mathbf{G}\mathbf{G}'}$ components ($\mathbf{G}\neq\mathbf{G}'$) from this expression. Incidentally, the rightmost argument of the function $G_0(z,z',\varepsilon)$ in Eq. (S16) refers to the out-of-plane electron energy $\hbar\varepsilon$ relative to the gold CBB, and consequently, the sums over i and $\mathbf{G}$ need to be restricted to only yield positive values of such argument.

We proceed by using the fact that the initial graphene states i are spatially localized near the $z=d$ plane [i.e., the out-of-plane spatial extension of $\varphi_i(z)$ ($\sim\pm1$ Å [25]) is small compared with the tunneling distance $d\sim 2-3$ nm], and therefore, Eq. (S16) can be approximated by setting $z'=z''=d$. In addition, the initial wave vectors $\mathbf{Q}_i$ relative to the graphene K_g point (see Sec. S6 B) are small compared with $\mathbf{K}_g$, and their energies $\hbar\varepsilon_i$ close to $E_{\rm F}^{\rm g}$, so we approximate $\varepsilon_i-\omega-\hbar|\mathbf{Q}_i-\mathbf{k}_\parallel+\mathbf{G}-\mathbf{K}_g|^2/2m_{\rm e}\approx E_{\rm F}^{\rm g}/\hbar-\omega-\hbar|\mathbf{G}-\mathbf{K}_g-\mathbf{k}_\parallel|^2/2m_{\rm e}$. The sum over i is then trivially yielding an overall constant. Putting these elements together, we transform Eq. (S16) into

$$\begin{aligned} J^{\rm ind-ex} \propto& \sum_{\mathbf{G}}\int_0^{eV_{\rm b}/\hbar} d\omega\int_{\rm 1BZ} d^2\mathbf{k}_\parallel\;{\rm Im}\big\{-W_{\mathbf{G}\mathbf{G}}(\mathbf{k}_\parallel,d,d,\omega)\big\} \\ &\times {\rm Im}\Big\{G_0^*(0,d,E_{\rm F}^{\rm g}/\hbar-\omega-\hbar|\mathbf{G}-\mathbf{K}_g-\mathbf{k}_\parallel|^2/2m_{\rm e}) \\ &\quad\times \partial_z G_0(z,d,E_{\rm F}^{\rm g}/\hbar-\omega-\hbar|\mathbf{G}-\mathbf{K}_g-\mathbf{k}_\parallel|^2/2m_{\rm e})\big|_{z=0^-}\Big\}. \end{aligned} \tag{S17}$$

Finally, we argue that $G_0(0,d,\varepsilon)$ is a strongly decreasing function of ε, and therefore, the lowest values of the argument ε should contribute maximally to the tunneling current. Such values are encountered at $\hbar\omega=eV_{\rm b}$ in Eq. (S17). Using an analogous argument, the region near the point $\mathbf{k}_\parallel=\mathbf{G}-\mathbf{K}_g$ for each reciprocal lattice vector $\mathbf{G}$ should produce a dominant contribution to the $\mathbf{k}_\parallel$ integral. In addition, because the relative orientation of the TMD and graphene lattices is undefined, we average over the azimuthal angle of $\mathbf{K}_g$, which we denote as $\varphi_{\mathbf{K}_g}$. By doing so, the tunneling current is found to depend on bias voltage $V_{\rm b}$ roughly as

$$\begin{aligned} J^{\rm ind-ex} &\propto \sum_{\mathbf{G}}\int_{\rm 1BZ} d^2\mathbf{k}_\parallel\;{\rm Im}\big\{-W_{\mathbf{G}\mathbf{G}}(\mathbf{k}_\parallel,d,d,eV_{\rm b}/\hbar)\big\}\int d\varphi_{\mathbf{K}_g}\;\delta(\mathbf{G}-\mathbf{K}_g-\mathbf{k}_\parallel) \\ &\propto \sum_{\mathbf{G}}\int_{\rm 1BZ} d^2\mathbf{k}_\parallel\;{\rm Im}\big\{-W_{\mathbf{G}\mathbf{G}}(\mathbf{k}_\parallel,d,d,eV_{\rm b}/\hbar)\big\}\;\delta\big(K_g-|\mathbf{G}-\mathbf{k}_\parallel|\big). \end{aligned} \tag{S18}$$

The 1BZ integration region of Eq. (S18) is represented in $\mathbf{k}_\parallel$ space in Fig. S12 for $MoSe_2$, while similar results are found for the other TMDs. The six nearest-neighbor TMD Brillouin zones are indicated by hexagons in this figure, each of them centered around its corresponding $\mathbf{G}$ vector. We also show the circles defined by the respective conditions $|\mathbf{G}-\mathbf{k}_\parallel|=K_g$. It is clear that only the six smallest non-vanishing TMD reciprocal lattice vectors $\mathbf{G}$ can satisfy such conditions for $\mathbf{k}_\parallel$ within the 1BZ. Furthermore, as we have averaged over the orientation of $\Gamma\mathrm{K}_g$ in graphene, and in virtue of symmetry, all of those $\mathbf{G}$'s should make equal contributions. Consequently, we only need to consider a single $\mathbf{G}=\mathbf{G}_0\equiv G_0\,\hat{\mathbf{x}}$ with $G_0=4\pi/(\sqrt{3}a_{\rm TMD})$, where $a_{\rm TMD}$ is the in-plane lattice constant of the TMD. This allows us to rewrite Eq. (S18) as

$$J^{\rm ind-ex}\propto\int_{G_0-K_g}^{G_0-\sqrt{3}K/2}\frac{dk_x}{k_y}\;{\rm Im}\big\{-W_{\mathbf{G}_0\mathbf{G}_0}(\mathbf{k}_\parallel,d,d,eV_{\rm b}/\hbar)\big\}, \tag{S19}$$

where $\mathbf{k}_\parallel = (k_x, k_y)$ with $k_y = \sqrt{K_g^2 - (G_0 - k_x)^2}$, and we recall that $K_g \approx 17.02$ nm^{-1} is the ΓK$_g$ distance in graphene. The integration contour in Eq. (S19) is indicated by a thick circular segment inside the 1BZ of Fig. S12.

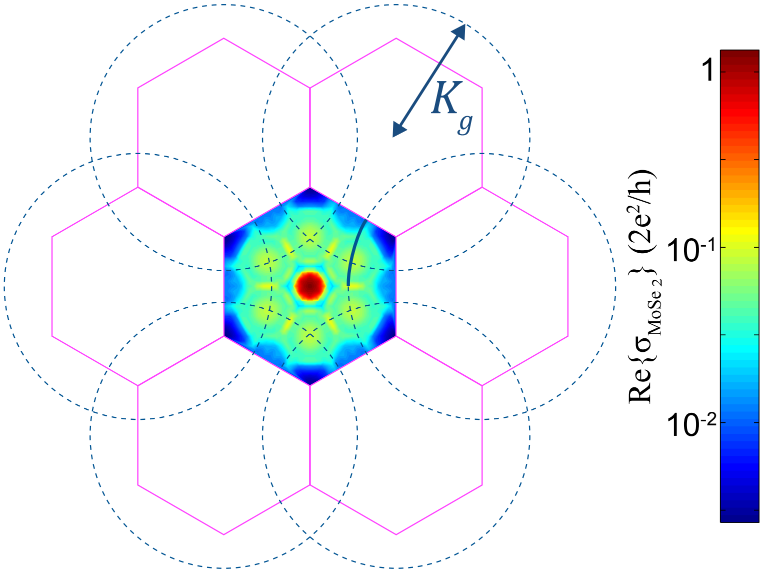

FIG. S12. **Leading exciton contributions to the tunneling current.** We plot Re$\{\sigma_{\rm TMD,00}(\mathbf{k}_\parallel, \omega)\}$ (i.e., the real part of the optical conductivity for $\mathbf{G} = \mathbf{G}' = 0$, see Secs. S8 and S9) within the 1BZ of MoSe$_2$ at a photon energy $\hbar\omega = 2.1$ eV, along with the six nearest-neighbor Brillouin zones (hexagons), each of them centered around a $\mathbf{G}$ vector. We also show the corresponding circles defined by the conditions $|\mathbf{G} - \mathbf{k}_\parallel| = K_g$ (i.e., the ΓK$_g$ distance in graphene).

The optical conductivity of the TMD, illustrated for MoSe$_2$ at $\hbar\omega = 2.1$ eV in Fig. S12 through a color plot of Re$\{\sigma_{\rm TMD,00}(\mathbf{k}_\parallel, \omega)\}$ (i.e., with $\mathbf{G} = \mathbf{G}' = 0$, see Secs. S8 and S9), shows that the response is dominated by localized momentum regions corresponding to the KQ$_{\rm TMD}$ excitons (see Table S2), inside of which the wave vector component k_y does not vary significantly, so we approximate the current as

$$J^{\rm ind-ex} \propto {\rm Im}\big\{ - W_{\mathbf{G}_0\mathbf{G}_0}(\mathbf{k}_\parallel, d, d, eV_{\rm b}/\hbar)\big\} \tag{S20}$$

for the sake of the discussion of one-step tunneling processes in the main text. [Nevertheless, the results presented in Fig. S13 and Fig. 4c in the main text are obtained by evaluating Eq. (S19).] We remind that $\mathbf{k}_\parallel$ is understood to be determined by the condition $|G_0\,\hat{\mathbf{x}} - \mathbf{k}_\parallel| = K_g$ in Eq. (S20).

We evaluate Eq. (S19) from the diagonal elements of the screened interaction $W_{\mathbf{GG}}(\mathbf{k}_\parallel, d, d, \omega)$, which are in turn calculated as explained in Sec. S8 below. A result for the I–V curve is presented in Fig. S13 for MoSe$_2$ with a hBN tunnel barrier distance $d = 3$ nm. Spectral features are clearly visible in the 1.9–2.2 eV range, corresponding to indirect excitons that are also observed in our first-principles calculations of the TMD conductivity (see Fig. S19 in Sec. S9). The spectral range in which these features show up is in good correspondence with experiments, although the detailed spectral shape presents differences that are possibly connected to the approximations adopted within the present theoretical model.

E. Two-step tunneling assisted by phonon and exciton creation

We now analyze two-step processes involving phonon excitation followed by the creation of nearly direct excitons as an additional channel contributing to electron tunneling from graphene to gold and also producing an excitonic signature in the dI/dV curves.

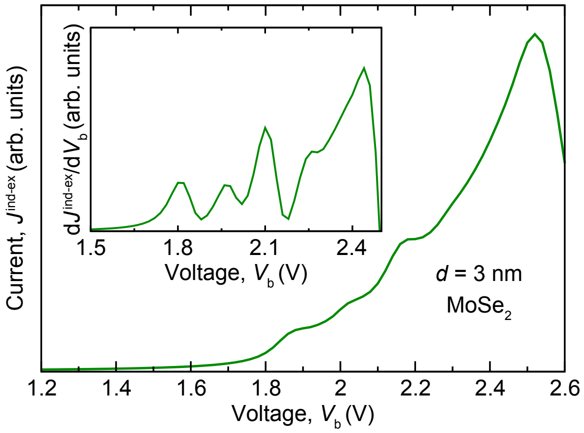

FIG. S13. **Calculated I–V curve for indirect exciton-assisted one-step tunneling.** We plot the current obtained by using Eq. (S20) for $MoSe_2$ (see Fig. 4 in the main text for other TMDs) with a hBN tunnel barrier distance $d = 3$ nm. The inset shows the corresponding dI/dV curve. Incidentally, the current drops beyond ~ 2.5 eV because of the reduced number of bands used to calculate the response associated with indirect excitons (see Sec. S9).

1. *Quasi-elastic phonon-assisted transitions*

The phonon energies at the K point are large compared with k_BT at room temperature T, and therefore, we can safely neglect phonon absorption processes. Starting from a graphene state i (see Sec. S6 B), within first-order perturbation theory, the excitation of a phonon p of wave vector $\mathbf{K}_g + \mathbf{Q}_p$ (i.e., with $\mathbf{Q}_i$ relative to the graphene K_g point) and frequency ω_p contributes with an electron wave function $\tilde{\varphi}_{ip}(\mathbf{r}) = \varphi_i(z)\,e^{i(\mathbf{Q}_i-\mathbf{Q}_p)\cdot\mathbf{R}}/\sqrt{A}$ of energy $\hbar(\varepsilon_i - \omega_p)$, where

$$\tilde{\varphi}_{ip}(\mathbf{r}) = \int d^3\mathbf{r}' G_0(\mathbf{r},\mathbf{r}',\varepsilon_i-\omega_p)\,\mathcal{H}_p^{\rm ph}(\mathbf{r}')\,\varphi_i(\mathbf{r}') \tag{S21}$$

is expressed in terms of the electron-phonon coupling Hamiltonian $\mathcal{H}_p^{\rm ph}(\mathbf{r}')$ and the 3D electron Green function $G_0(\mathbf{r},\mathbf{r}',\varepsilon) = (2\pi)^{-1}\int d^2\mathbf{Q}\,e^{i\mathbf{Q}\cdot(\mathbf{R}-\mathbf{R}')}\,G_0(z,z',\varepsilon-\hbar Q^2/2m_e)$ [see Sec. S6 B for details of this function and the definitions of $\varphi_i(z)$, $\mathbf{Q}_i$, and A]. Separating the in- and out-of-plane dependence of the phonon Hamiltonian as $\mathcal{H}_p^{\rm ph}(\mathbf{r}') = \mathcal{H}_p^{\rm ph}(z')e^{-i(\mathbf{K}_g+\mathbf{Q}_p)\cdot\mathbf{R}}$, we can recast Eq. (S21) into

$$\tilde{\varphi}_{ip}(z) = \int dz' G_0\big(z,z',\varepsilon_i-\omega_p-\hbar|\mathbf{Q}_i-\mathbf{Q}_p|^2/2m_e\big)\,\mathcal{H}_p^{\rm ph}(z')\,\varphi_i(z'),$$

and noticing again that $\varphi_i(z)$ is tightly localized around $z = d$, we can approximate this expression as

$$\tilde{\varphi}_{ip}(z) \approx C_p\,G_0\big(z,d,\varepsilon_i-\omega_p-\hbar|\mathbf{Q}_i-\mathbf{Q}_p|^2/2m_e\big), \tag{S22}$$

where the constant C_p depends on the specific phonon mode p under consideration. In addition, as tunneling is expected to involve large phonon momentum transfers that place the electron near the Γ point, only a reduced region in the phonon bands contributes to the process, and therefore, we assume $C_1 \equiv |C_p|$ to be roughly independent of p. The tunneling current per unit area traversing the hBN/Au interface is finally obtained as

$$\begin{aligned} J^{\rm ph} &= -\frac{e\hbar}{m_e}\sum_{ip}\Theta\big[\hbar(\varepsilon_i-\omega_p)-E_F^{\rm Au}\big]\,{\rm Im}\big\{\tilde{\varphi}_{ip}^*(\mathbf{r})\partial_z\tilde{\varphi}_{ip}(\mathbf{r})\big\}\big|_{z=0^-} \\ &\approx -\frac{C_1^2}{A}\frac{e\hbar}{m_e}\sum_{ip}\Theta\big[\hbar(\varepsilon_i-\omega_p)-E_F^{\rm Au}\big]\,{\rm Im}\Big\{G_0^*\big(z,d,\varepsilon_i-\omega_p-\hbar|\mathbf{Q}_i-\mathbf{Q}_p|^2/2m_e\big) \\ &\qquad\qquad \times\,\partial_z G_0\big(z,d,\varepsilon_i-\omega_p-\hbar|\mathbf{Q}_i-\mathbf{Q}_p|^2/2m_e\big)\Big\}\Big|_{z=0^-}, \end{aligned}$$

which is evaluated at a position $z = 0^-$ right inside the metal (see Fig. S11 and Sec. S6 C), with the step function restricting the sum over i and p such that only final unoccupied electron gold states above the Fermi level $E_{\rm F}^{\rm Au}$ are included.

2. *Phonon+exciton-assisted tunneling*

In the two-step channel, after a phonon has been created, a direct exciton absorbs most of the energy lost by the tunneling electron. This second step is described by Eq. (S12) in Sec. S6 C, with φ_i substituted by the phonon-scattered wave function $\tilde{\varphi}_{ip}$ given in Eq. (S22). Following a similar procedure as in Sec. S6 D, we insert Eqs. (S3) and (S14) into Eq. (S12) and carry out the integrals over $\mathbf{R}$, $\mathbf{R}'$, and $\mathbf{R}''$ to find

$$J^{\rm ph+ex} = \frac{1}{A}\frac{e^3\hbar^2}{\pi m_{\rm e}}\sum_{ip}\int_0^\infty d\omega\, \Theta\big[\hbar(\varepsilon_i-\omega_p-\omega)-E_{\rm F}^{\rm Au}\big]\int\frac{d^2\mathbf{k}_\parallel}{(2\pi)^2}\int dz'\int dz''\,{\rm Im}\big\{-W(k_\parallel,z',z'',\omega)\big\}$$
$$\times\,\Theta(\varOmega_{ip\omega\mathbf{k}_\parallel})\,{\rm Im}\Big\{G_0^*(0,z',\varOmega_{ip\omega\mathbf{k}_\parallel})\,\partial_z G_0(z,z'',\varOmega_{ip\omega\mathbf{k}_\parallel})\big|_{z=0^-}\,\tilde{\varphi}_{ip}^*(z')\tilde{\varphi}_{ip}(z'')\Big\}, \qquad \text{(S23)}$$

where we have assumed translational invariance in the screened interaction [Eq. (S14)] and defined $\varOmega_{ip\omega\mathbf{k}_\parallel} = \varepsilon_i - \omega_p - \omega - \hbar|\mathbf{Q}_i - \mathbf{Q}_p - \mathbf{k}_\parallel|^2/2m_{\rm e}$.

We note that the intermediate phonon-assisted state $\tilde{\varphi}_{ip}(z)$ is generated from the graphene region (at $z = d$), and therefore, we expect it to be exposed to further inelastic interactions from that region, including TMD excitons. Rather than propagating it elastically (once the phonon has been created) to tunnel into the metal (i.e., like in Sec. S6 E 1), we are interested in the immediate interaction with the excitons. Therefore, proceeding as we did in Sec. S6 D, we approximate Eq. (S23) by setting $z' = z'' = d$ and encapsulating the dependence on the intermediate states in an overall constant factor, so we write

$$J^{\rm ph+ex} \propto \sum_{ip}\int_0^\infty d\omega\,\Theta\big[\hbar(\varepsilon_i-\omega_p-\omega)-E_{\rm F}^{\rm Au}\big]\int d^2\mathbf{k}_\parallel\,{\rm Im}\big\{-W(k_\parallel,d,d,\omega)\big\} \qquad \text{(S24)}$$
$$\times\,{\rm Im}\Big\{G_0^*(0,d,\varepsilon_i-\omega_p-\omega-\hbar|\mathbf{Q}_i-\mathbf{Q}_p-\mathbf{k}_\parallel|^2/2m_{\rm e})$$
$$\times\,\partial_z G_0(z,d,\varepsilon_i-\omega_p-\omega-\hbar|\mathbf{Q}_i-\mathbf{Q}_p-\mathbf{k}_\parallel|^2/2m_{\rm e})\big|_{z=0^-}\Big\}.$$

We further simplify the evaluation of Eq. (S24) by arguing that the response function dies out quickly as $k_\parallel$ increases to values beyond 1 nm^{-1} (see Sec. S7 and Fig. S18), which is small compared with both $K_g \approx 17.02$ nm^{-1} and $k_{\rm F}^{\rm Au} \approx 12$ nm^{-1}, so we can dismiss the $\mathbf{k}_\parallel$ dependence in front of $\mathbf{Q}_p$ in the last argument of the Green function G_0. Likewise, the wave vectors of graphene electrons relative to the Dirac point are also small and can be neglected in G_0 when compared with $\mathbf{Q}_p$. We thus approximate $|\mathbf{Q}_i - \mathbf{Q}_p - \mathbf{k}_\parallel| \approx Q_p$. In contrast, the phonon wave vector $\mathbf{Q}_p$ is large, so it has to be retained. We replace the sum over phonons p by $C_2\int_0^\infty Q dQ$, where $Q \equiv Q_p$ and the constant C_2 accounts for further details of the electron-phonon coupling. Analogously, we replace the sum over graphene electrons i by $C_3\sum_{s=\pm1}\int_0^{Q_s} Q'dQ'$, where $Q' \equiv Q_i$ and the constant C_3 incorporates further graphene band details. We also sum over $s = +1$ and $s = -1$, referring to the upper (with Q' running up to the graphene Fermi wave vector $Q_1 = k_{\rm F}^{\rm g}$) and lower (with $Q_{-1} = \infty$) Dirac cones, respectively. In addition, we assume ω_p to be independent of p. These considerations allow us to reduce Eq. (S24) to

$$J^{\rm ph+ex} \propto \sum_{s=\pm1}\int_0^\infty d\omega\int_0^{Q_s} Q'dQ'\,\Theta\big[\hbar(\varepsilon_0+sv_{\rm F}^{\rm g}Q'-\omega_p-\omega)-E_{\rm F}^{\rm Au}\big]\int_0^\infty k_\parallel dk_\parallel\,{\rm Im}\big\{-W(k_\parallel,d,d,\omega)\big\}$$
$$\times\int_0^{Q_c} QdQ\,{\rm Im}\Big\{G_0^*(0,d,\varepsilon_0+sv_{\rm F}^{\rm g}Q'-\omega_p-\omega-\hbar Q^2/2m_{\rm e})$$
$$\times\,\partial_z G_0(z,d,\varepsilon_0+sv_{\rm F}^{\rm g}Q'-\omega_p-\omega-\hbar Q^2/2m_{\rm e})\big|_{z=0^-}\Big\}, \qquad \text{(S25)}$$

where $\hbar\varepsilon_0 \equiv E_{\rm D} = E_{\rm F}^{\rm g} - \varDelta E_{\rm F}^{\rm g}$ is the Dirac point energy relative to the CBB of gold (see Figs. S10 and S11), and a cutoff wave vector $Q_c = \big[2m_{\rm e}(\varepsilon_0 + sv_{\rm F}^{\rm g}Q' - \omega_p - \omega)/\hbar\big]^{1/2}$ is introduced to guarantee that the final out-of-plane electron energy is above the CBB of gold.

We are interested in examining the tunneling current for bias potential energies close to the TMD exciton energies, which are large compared with the graphene electron energies relative to the Dirac point. Therefore, we further

approximate Eq. (S25) by setting $\varepsilon_0 + s v_F^g Q' \approx E_F^g/\hbar = (E_F^{Au} + eV_b)/\hbar \equiv \varepsilon_1$ and treating $\sum_s \int Q' dQ'$ as an additional overall constant. This leads to

$$J^{ph+ex} \propto \int_0^{eV_b/\hbar - \omega_p} d\omega \int_0^\infty k_\parallel dk_\parallel \, \mathrm{Im}\{-W(k_\parallel, d, d, \omega)\} \times \int_0^{Q_c} Q dQ \, \mathrm{Im}\Big\{G_0^*(0, d, \varepsilon_1 - \omega_p - \omega - \hbar Q^2/2m_e)\, \partial_z G_0(z, d, \varepsilon_1 - \omega_p - \omega - \hbar Q^2/2m_e)\big|_{z=0^-}\Big\}, \tag{S26}$$

where we need to redefine $Q_c = \big[2m_e(\varepsilon_1 - \omega_p - \omega)/\hbar\big]^{1/2}$.

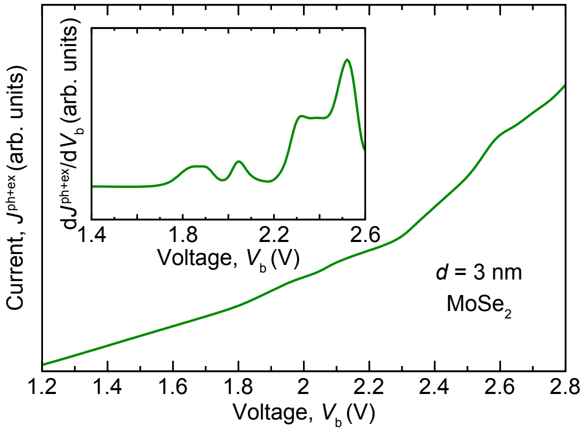

FIG. S14. **Calculated I–V curves for phonon+exciton-assisted two-step tunneling.** We plot the current obtained by using Eq. (S27) for $MoSe_2$ (see Fig. 4 in the main text for other TMDs) with a hBN tunnel barrier distance $d = 3$ nm. The inset shows the corresponding dI/dV curve.

Finally, we argue that $G_0(0, d, \varepsilon)$ is a strongly decreasing function of ε, and therefore, the lowest values of the argument ε should contribute maximally to the tunneling current. Such values are encountered at $\hbar\omega = eV_b$ according to Eq. (S26), from which the phonon+exciton-assisted tunneling current is found to depend on bias voltage V_b roughly as

$$J^{ph+ex} \propto \int_0^\infty k_\parallel dk_\parallel \, \mathrm{Im}\{-W(k_\parallel, d, d, eV_b/\hbar - \omega_p)\}. \tag{S27}$$

This result relates the phonon+exciton-assisted tunneling current to the screened interaction in the heterostructure. The latter is studied in detail in Sec. S7.

In Fig. S14, we plot the I–V curves obtained from Eq. (S27) for $d = 3$ nm, with the screened interaction obtained by describing gold in the specular-reflection model, hBN through a local anisotropic permittivity, graphene in the random-phase approximation (RPA), and the TMD monolayer through a nonlocal surface conductivity (see Sec. S9). The tunneling current shows dominant features associated with C excitons, while A and B excitons emerge as weak shoulders at lower biases.

Upon examination of the $k_\parallel$ dependence of $\mathrm{Im}\{-W(k_\parallel, d, d, eV_b/\hbar - \omega_p)\}$ (see Fig. S18), we conclude that the integral in Eq. (S27) yields a result similar to $\mathrm{Im}\{-W(0, d, d, eV_b/\hbar - \omega_p)\}$ (i.e., at $k_\parallel = 0$), so we write

$$J^{ph+ex} \propto \mathrm{Im}\{-W_{00}(0, d, d, eV_b/\hbar - \omega_p)\},$$

where we insert subindices indicating that this part involves $\mathbf{G} = 0$ (i.e., no umklapp processes), for the sake of the discussion of two-step tunneling in the main text. [Nevertheless, the results presented in Fig. S14 and Fig. 4d in the main text are obtained by evaluating Eq. (S27).]

S7. Screened interaction under the in-plane isotropic and translational-invariant approximations

Tunneling electrons couple to excitations in the structure through the screened interaction $W(\mathbf{r}, \mathbf{r}', \omega)$. Because the distances involved are small compared with the optical wavelength at frequency ω, we can safely neglect retardation

effects and treat the interaction in the electrostatic limit. The structure under consideration is sketched in Fig. S15a and consists of a layer of 2D materials separated by a distance d from a gold surface. The region in between the two of them is filled with hBN, and the medium above the 2D materials is also hBN.

The layer of 2D materials here considered consists of a TMD monolayer on top of graphene. We describe it in the zero-thickness approximation through a nonlocal wave-vector- and frequency-dependent surface conductivity $\sigma(k_\parallel,\omega)$. This approximation simplifies the algebra considerably and yields reasonable results in comparison to finite-thickness layers [26]. We thus set $\sigma(k_\parallel,\omega)=\sigma_{\rm TMD}(k_\parallel,\omega)+\sigma_{\rm g}(k_\parallel,\omega)$ as the sum of the surface conductivities of the TMD and graphene monolayers. A nonlocal version of the former is calculated from first principles (see Sec. S9), whereas the nonlocal conductivity of graphene is modeled in the RPA [27].

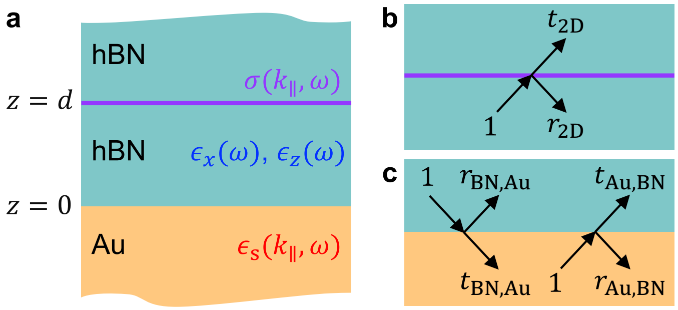

FIG. S15. **Analysis of the dielectric response of the studied multilayer structure.** (**a**) We consider a 2D material described by a nonlocal surface conductivity $\sigma(k_\parallel,\omega)$, separated from a gold surface by an hBN tunnel barrier of thickness d. The structure is covered by an upper hBN layer. Gold is described through a surface-corrected nonlocal dielectric function $\epsilon_{\rm s}(k_\parallel,\omega)$, whereas hBN is modeled through its anisotropic frequency-dependent permittivity [28] of in-plane and out-of-plane components $\epsilon_x(\omega)$ and $\epsilon_z(\omega)$, respectively. The gold and upper hBN media are assumed to have an infinite thickness, while the 2D material is treated in the zero-thickness limit. (**b,c**) The response of the system is described in terms of Fresnel transmission and reflection coefficients for each of the two interfaces.

The interfaces in our structure are described by first defining Fresnel's reflection coefficients, as shown in Fig. S15b,c. More precisely, only p-polarization coefficients survive in the electrostatic limit. For convenience, we define in-plane coordinates $\mathbf{R}=(x,y)$ and work in the space of in-plane wave vectors $\mathbf{k}_\parallel=(k_x,k_y)$, with electrostatic quantities such as the electric potential written as $\phi(\mathbf{r},\omega)=(2\pi)^{-2}\int d^2\mathbf{k}_\parallel\,{\rm e}^{{\rm i}\mathbf{k}_\parallel\cdot\mathbf{R}}\,\phi(\mathbf{k}_\parallel,z,\omega)$.

We start by considering the electrostatic potential created by a point charge of time-dependent magnitude ${\rm e}^{-{\rm i}\omega t}$ placed at the origin in bulk hBN. The momentum components of such potential are given by $[2\pi/k_\parallel\bar\epsilon(\omega)]\,{\rm e}^{-q|z|}$, where $\bar\epsilon(\omega)=\sqrt{\epsilon_x(\omega)\epsilon_z(\omega)}$ and $q=k_\parallel\sqrt{\epsilon_x(\omega)/\epsilon_z(\omega)}$, with the square root sign chosen to yield positive real parts. Now, with the 2D material layer placed at $z=d$, scattering of an *incident* potential component ${\rm e}^{-q(z-d)}$ coming from the $z<d$ region gives rise to a *transmitted* potential $t_{\rm 2D}{\rm e}^{-q(z-d)}$ at $z>d$ and an *incident+reflected* potential ${\rm e}^{-q(z-d)}-r_{\rm 2D}{\rm e}^{q(z-d)}$ at $z<d$. (Incidentally, note that the reflection coefficient is preceded by a $-$ sign in this expression for the potential [26].) The corresponding Fresnel coefficients are determined by imposing the continuity of the potential at $z=d$, as well as the condition that the jump in the normal electric displacement is 4π times the surface charge produced through the conductivity $\sigma(k_\parallel,\omega)$. Writing such charge in terms of the current through the continuity equation, and this in turn as the conductivity times the in-plane electric field, we are left with a set of two linear equations from which we obtain

$$r_{\rm 2D}=\frac{1}{1-{\rm i}\omega\bar\epsilon(\omega)/[2\pi k_\parallel\sigma(k_\parallel,\omega)]}, \tag{S28a}$$

$$t_{\rm 2D}=1-r_{\rm 2D} \tag{S28b}$$

for the Fresnel coefficients of the 2D layer, which generalize previous results [29] to deal with an anisotropic host medium. We note that the z dependence of the potential components inside hBN is simply given by ${\rm e}^{\mp q(z-d)}$ for upward ($-$ sign) and downward ($+$ sign) components (see Fig. S15b).

Given the small thickness of the hBN tunnel layer, nonlocal effects in the response of gold can play an important role, and therefore, we use the nonlocal bulk permittivity of this material $\epsilon_{\rm Au}(k,\omega)$. As a reasonably accurate prescription, we express $\epsilon_{\rm Au}(k,\omega)$ as the sum of a conduction-electron component, treated in the RPA, and a local contribution associated with interband transitions, as described in detail elsewhere [30]. In addition, we adopt the

specular-reflection model [23, 30, 31] to incorporate surface effects through a surface response function

$$\epsilon_{\rm s}(k_\parallel,z,\omega)=\frac{k_\parallel}{\pi}\int\frac{dk_z}{k^2}\frac{{\rm e}^{{\rm i}k_zz}}{\epsilon_{\rm Au}(k,\omega)}=\frac{2k_\parallel}{\pi}\int_0^\infty\frac{dk_z}{k^2}\frac{\cos(k_zz)}{\epsilon_{\rm Au}(k,\omega)},$$

where $k^2=k_\parallel^2+k_z^2$. Incidentally, this quantity becomes $\epsilon_{\rm s}(k_\parallel,z,\omega)={\rm e}^{-k_\parallel|z|}/\epsilon_{\rm Au}(\omega)$ in the local limit (i.e., when dismissing the k dependence of the gold permittivity). We also define $\epsilon_{\rm s}(k_\parallel,\omega)\equiv\epsilon_{\rm s}(k_\parallel,0,\omega)$, in terms of which we find the Fresnel coefficients

$$r_{\rm Au,BN}=-r_{\rm BN,Au}=\frac{\bar\epsilon(\omega)\epsilon_{\rm s}(k_\parallel,\omega)-1}{\bar\epsilon(\omega)\epsilon_{\rm s}(k_\parallel,\omega)+1},\tag{S29a}$$

$$t_{\rm Au,BN}=\frac{2}{\bar\epsilon(\omega)\epsilon_{\rm s}(k_\parallel,\omega)+1},\tag{S29b}$$

$$t_{\rm BN,Au}=\frac{2\,\bar\epsilon(\omega)\epsilon_{\rm s}(k_\parallel,\omega)}{\bar\epsilon(\omega)\epsilon_{\rm s}(k_\parallel,\omega)+1}\tag{S29c}$$

for the hBN/Au interface by imposing the continuity of both the potential and the normal electric displacement. Incidentally, the latter involves $\epsilon_{\rm Au}\cdot\epsilon_{\rm s}$ (in operator notation), which reduces to ${\rm e}^{-k_\parallel|z|}$ in $(k_\parallel,z)$ space. With the hBN/Au interface placed at $z=0$, the z dependence of the potential indicated by the arrows in Fig. S15c is given by ${\rm e}^{\mp qz}$ for $z>0$. However, the dependence inside gold ($z<0$) is more complex [23]: it is partially encapsulated in a factor $\epsilon_{\rm s}(k_\parallel,z,\omega)/\epsilon_{\rm s}(k_\parallel,\omega)$ multiplying the transmission coefficients; in addition, an involved dependence is encountered for internal reflection from the gold side (see more details in Ref. [23]).

Finally, the screened interaction can also be decomposed in parallel wave vectors as shown in Eq. (S14), where $W(k_\parallel,z,z',\omega)=W^{\rm dir}(k_\parallel,z,z',\omega)+W^{\rm ref}(k_\parallel,z,z',\omega)$ is in turn separated into direct and reflected components. The former corresponds to the direct Coulomb interaction when z and z' lie in the same medium (see Fig. S15a):

$$W^{\rm dir}(k_\parallel,z,z',\omega)=\frac{2\pi}{k_\parallel}\times\begin{cases}\dfrac{1}{\bar\epsilon(\omega)}{\rm e}^{-q|z-z'|}, & z,z'\geq0\text{ inside hBN},\\ \epsilon_{\rm s}(k_\parallel,z-z',\omega), & z,z'<0\text{ inside Au},\\ 0, & \text{otherwise},\end{cases}\tag{S30}$$

where the part inside gold incorporates nonlocal bulk corrections. The remaining surface contribution is given by

$$W^{\rm ref}(k_\parallel,z,z',\omega)=\frac{2\pi}{k_\parallel}\times\begin{cases}-\dfrac{1}{\bar\epsilon(\omega)}{\rm e}^{-q(z+z'-2d)}\left[r_{\rm 2D}+{\rm e}^{-2qd}\varDelta\,t_{\rm 2D}^2r_{\rm BN,Au}\right], & d\leq z,z',\\ \dfrac{1}{\bar\epsilon(\omega)}\varDelta\,t_{\rm 2D}\left[{\rm e}^{-q(z-z')}-{\rm e}^{-q(z+z')}r_{\rm BN,Au}\right]-\dfrac{{\rm e}^{-q(z-z')}}{\bar\epsilon(\omega)}, & 0\leq z'<d\leq z,\\ {\rm e}^{-qz}\varDelta\,t_{\rm 2D}t_{\rm Au,BN}\epsilon_{\rm s}(k_\parallel,z',\omega), & d\leq z,\ z'<0,\\ \dfrac{1}{\bar\epsilon(\omega)}\varDelta\left[\left({\rm e}^{-q(2d+z-z')}+{\rm e}^{-q(2d-z+z')}\right)r_{\rm 2D}r_{\rm BN,Au}\right.\\ \qquad\left.-{\rm e}^{-q(2d-z-z')}r_{\rm 2D}-{\rm e}^{-q(z+z')}r_{\rm BN,Au}\right], & 0\leq z,z'<d,\\ \varDelta\,t_{\rm Au,BN}\left[{\rm e}^{-qz}-{\rm e}^{-q(2d-z)}r_{\rm 2D}\right]\epsilon_{\rm s}(k_\parallel,z',\omega), & z'<0\leq z<d,\\ \epsilon_{\rm s}(k_\parallel,z+z',\omega)-\bar\epsilon(\omega)\varDelta\,t_{\rm Au,BN}\left[1+{\rm e}^{-2qd}r_{\rm 2D}\right]\\ \qquad\times\,\epsilon_{\rm s}(k_\parallel,z,\omega)\epsilon_{\rm s}(k_\parallel,z',\omega), & z,z'<0,\end{cases}\tag{S31}$$

for $z\geq z'$, while the reciprocity property $W^{\rm dir}(k_\parallel,z,z',\omega)=W^{\rm dir}(k_\parallel,z',z,\omega)$ can be used to compute it for $z<z'$. In Eq. (S31), we use the Fabry–Perot factor $\varDelta=\left(1-r_{\rm 2D}r_{\rm BN,Au}{\rm e}^{-2qd}\right)^{-1}$ to account for multiple round trips within the 2D/hBN/Au cavity. We have explicitly verified that the sum of Eqs. (S30) and (S31) is continuous in both z and z' across the $z=0$ and $z=d$ interfaces, while the normal displacement is continuous at $z=0$ and undergoes a jump by $2\,\bar\epsilon\,k_\parallel/(1-1/r_{\rm 2D})={\rm i}\omega\bar\epsilon^2/\pi\sigma(k_\parallel,\omega)$ at $z=d$ due to the presence of the surface conductivity associated with the 2D materials.

S8. Screened interaction including the atomic lattice periodicity

A more rigorous description of the screened interaction incorporating the in-plane atomic corrugation of the TMD layer may become necessary when large wave vectors are involved, such as in the current experiment (i.e., for momentum transfers $\sim\Gamma{\rm K}_g$ in graphene). This is the case of the one-step tunneling channel mediated by the creation

of indirect excitons (see Sec. S6 D), as described by Eq. (S17), in which the screened interaction enters through the components $W_{\mathbf{GG}'}(\mathbf{k}_\parallel, d, d, \omega)$ [see Eq. (S15)], with the source and probing positions both lying on the $z = d$ plane (i.e., where the TMD–graphene heterostructure is located, assuming again a zero-thickness limit in the description of its optical response).

We obtain $W_{\mathbf{GG}'}(\mathbf{k}_\parallel, d, d, \omega)$ directly from a Fabry–Perot (FP) description of the pathways followed by the electric potential generated by a time-dependent unit charge of magnitude $\mathrm{e}^{-\mathrm{i}\omega t}$ placed at $\mathbf{r}'$ with $z' = d^+$ taken right above the 2D material layer. Using the notation and methods of Sec. S7, the direct potential in an infinite hBN host medium can be written in a form analogous to Eq. (S15):

$$\phi^{\rm dir}(\mathbf{r}, \omega) = \sum_{\mathbf{G}} \int_{\rm 1BZ} \frac{d^2\mathbf{k}_\parallel}{(2\pi)^2} \, \mathrm{e}^{\mathrm{i}(\mathbf{k}_\parallel + \mathbf{G})\cdot\mathbf{R}} \phi^{\rm dir}_{\mathbf{G}}(\mathbf{k}_\parallel, z, \omega),$$

where

$$\phi^{\rm dir}_{\mathbf{G}}(\mathbf{k}_\parallel, z, \omega) = \frac{2\pi}{|\mathbf{k}_\parallel + \mathbf{G}|\bar\epsilon} \, \mathrm{e}^{-q_{\mathbf{G}}|z-d|}$$

and we define $q_{\mathbf{G}} = |\mathbf{k}_\parallel + \mathbf{G}|\sqrt{\epsilon_x(\omega)/\epsilon_z(\omega)}$ similar to q in Sec. S7, but with $k_\parallel$ substituted by $|\mathbf{k}_\parallel + \mathbf{G}|$. We now substitute the reflection and transmission coefficients $r_{\rm 2D}$ and $t_{\rm 2D}$ of the 2D material layer by reflection and transmission tensors of components $r_{{\rm 2D},\mathbf{GG}'}$ and $t_{{\rm 2D},\mathbf{GG}'}$, labelled by incident ($\mathbf{G}'$) and reflected/transmitted ($\mathbf{G}$) reciprocal lattice vectors (see below). In addition, the reflection coefficient at the BN–Au interface is still described by Eq. (S29a), assuming isotropy and translational invariance (i.e., $\mathbf{k}_\parallel$ and $\mathbf{G}$ are both conserved upon reflection at this interface), but introducing a $\mathbf{G}$ dependence by also replacing $k_\parallel$ by $|\mathbf{k}_\parallel + \mathbf{G}|$ in such an equation, which we indicate by writing the corresponding reflection coefficient as $r_{{\rm BN,Au},\mathbf{G}}$. Using these elements, a straightforward FP analysis allows us to write

$$W_{\mathbf{GG}'}(\mathbf{k}_\parallel, d, d, \omega) = \frac{2\pi}{|\mathbf{k}_\parallel + \mathbf{G}'|\bar\epsilon}\left[\delta_{\mathbf{GG}'} - r_{{\rm 2D},\mathbf{GG}'} - \sum_{\mathbf{G}_1\mathbf{G}_2} t_{{\rm 2D},\mathbf{GG}_2} \, \mathrm{e}^{-q_{\mathbf{G}_2}d} \, \varDelta_{\mathbf{G}_2\mathbf{G}_1} \, r_{{\rm BN,Au},\mathbf{G}_1} \, \mathrm{e}^{-q_{\mathbf{G}_1}d} \, t_{{\rm 2D},\mathbf{G}_1\mathbf{G}'}\right], \quad \text{(S33)}$$

where $\varDelta$ is a tensor of inverse components $\left[\varDelta^{-1}\right]_{\mathbf{GG}'} = \delta_{\mathbf{GG}'} - r_{{\rm BN,Au},\mathbf{G}} \, r_{{\rm 2D},\mathbf{GG}'} \, \mathrm{e}^{-(q_{\mathbf{G}}+q_{\mathbf{G}'})d}$.

The reflection and transmission tensors of the 2D layer entering Eq. (S33) can be in turn obtained from the conductivity tensor $\sigma_{\mathbf{GG}'}(\mathbf{k}_\parallel, \omega)$ by applying the same boundary conditions as invoked in the derivation of Eqs. (S28), but taking into account the expansion of different quantities in reciprocal-lattice-vector components. A detailed analysis leads to the result

$$r_{{\rm 2D},\mathbf{GG}'} = \left[\frac{\mathcal{I}}{\mathcal{I} + 2\bar\epsilon\,\mathcal{A}^{-1}}\right]_{\mathbf{GG}'},$$
$$t_{{\rm 2D},\mathbf{GG}'} = \delta_{\mathbf{GG}'} - r_{{\rm 2D},\mathbf{GG}'},$$

where $\mathcal{I}_{\mathbf{GG}'} = \delta_{\mathbf{GG}'}$ is the identity matrix and we introduce a tensor of components

$$\mathcal{A}_{\mathbf{GG}'} = \frac{4\pi\mathrm{i}}{\omega} \, \frac{(\mathbf{k}_\parallel + \mathbf{G})\cdot(\mathbf{k}_\parallel + \mathbf{G}')}{|\mathbf{k}_\parallel + \mathbf{G}|} \, \sigma_{\mathbf{GG}'}(\mathbf{k}_\parallel, \omega). \quad \text{(S35)}$$

Obviously, the $\mathbf{G} = \mathbf{G}' = 0$ elements $r_{{\rm 2D},00}$ and $t_{{\rm 2D},00}$ reproduce Eqs. (S28).

Finally, we express the conductivity tensor $\sigma_{\mathbf{GG}'}(\mathbf{k}_\parallel, \omega)$ in terms of the non-interacting susceptibility tensor $\chi^0_{\mathbf{GG}'}(\mathbf{k}_\parallel, \omega)$ by writing the charge $\rho^{\rm ind}$ induced in the 2D layer in response to a total potential ϕ expressed in $(\mathbf{r}, \omega)$ space as $\rho^{\rm ind} = (\mathrm{i}\omega)^{-1}\nabla\cdot\mathbf{j}^{\rm ind} = (\mathrm{i}/\omega)\nabla\cdot\sigma\nabla * \phi$, where $\mathbf{j}^{\rm ind}$ is the induced current, given in turn by the conductivity times the electric field $-\nabla\phi$. Here, the dot stands for the scalar product of two 2D vectors and the star indicates a functional linear operation. By definition, the non-interacting susceptibility relates the total potential and the induced charge as $\rho^{\rm ind} = \chi^0 * \phi$, so we have the relation $\chi^0 = (\mathrm{i}/\omega)\nabla\cdot\sigma$. Projecting on $\mathbf{k}_\parallel$ (within the 1BZ) and $\mathbf{G}$ components, we obtain

$$\sigma_{\mathbf{GG}'}(\mathbf{k}_\parallel, \omega) = \frac{\mathrm{i}\omega}{(\mathbf{k}_\parallel + \mathbf{G})\cdot(\mathbf{k}_\parallel + \mathbf{G}')} \, \chi^0_{\mathbf{GG}'}(\mathbf{k}_\parallel, \omega). \quad \text{(S36)}$$

Incidentally, using Eq. (S36), we can write the matrix in Eq. (S35) as $\mathcal{A}_{\mathbf{GG}'} = -4\pi\,|\mathbf{k}_\parallel + \mathbf{G}|^{-1}\,\chi^0_{\mathbf{GG}'}(\mathbf{k}_\parallel, \omega)$.

As explained in Sec. S7, the conductivity of the TMD–graphene 2D heterostructure is taken as the sum $\sigma(\mathbf{k}_\parallel, \omega) = \sigma_{\rm TMD}(\mathbf{k}_\parallel, \omega) + \sigma_{\rm g}(\mathbf{k}_\parallel, \omega)$. We apply Eq. (S36) to obtain the conductivity of the TMD monolayer from the susceptibility

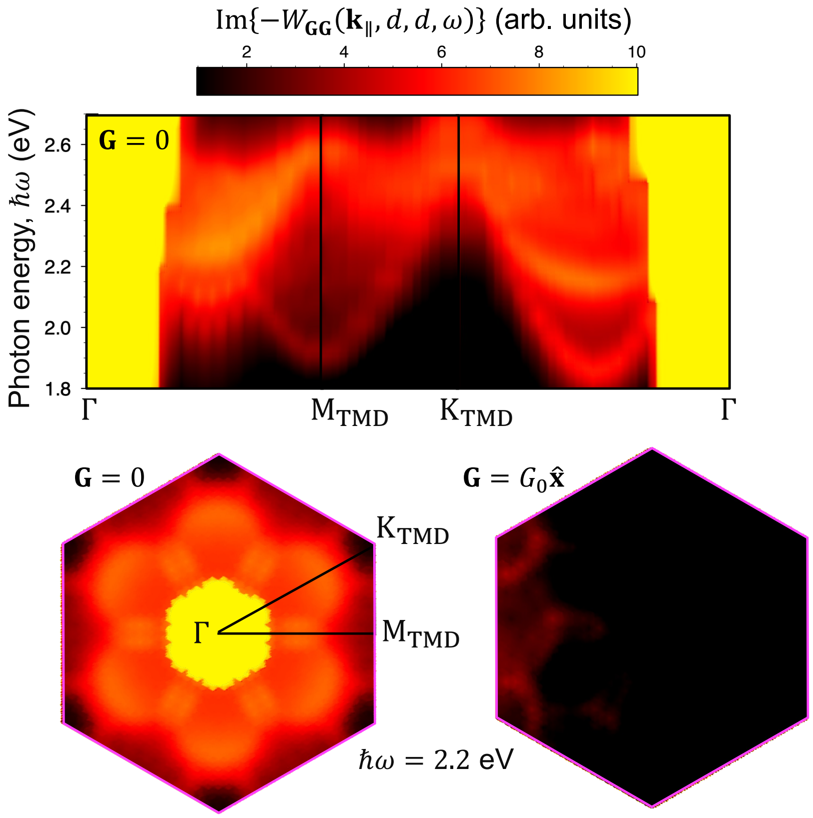

FIG. S16. **Screened interaction in $MoSe_2$.** We plot $\mathrm{Im}\{-W_{\mathbf{GG}}(\mathbf{k}_\parallel, d, d, \omega)\}$ for a heterostructure like that in Fig. S15 with $d = 3$ nm and the 2D layer consisting of monolayer $MoSe_2$ and graphene. The upper plot shows the dependence on photon energy and momentum along the indicated excursion within the 1BZ of the TMD for $\mathbf{G} = 0$. The lower plots show the $\mathbf{k}_\parallel$ dependence within the 1BZ for $\hbar\omega = 2.2$ eV and two values of $\mathbf{G}$ [0 and $G_0\hat{\mathbf{x}}$, with $G_0 = 4\pi/(\sqrt{3}a_{\rm TMD})$, where $a_{\rm TMD}$ is the in-plane lattice constant of the TMD]. The color scale is shared by all plots and saturated outside the displayed range.

calculated from first principles in Sec. S9. In addition, because the excitons involved in the tunneling process are supported by the TMD layer, and the 1BZ of graphene is substantially larger than that of the TMD, we treat the conductivity of graphene in the isotropic and translational-invariant approximations [i.e., $\sigma_{\rm g}(k_\parallel, \omega)$ is calculated in the RPA [27], as described in Sec. S7], with tensor components given by $\sigma_{{\rm g},\mathbf{GG'}}(\mathbf{k}_\parallel, \omega) = \delta_{\mathbf{GG'}}\, \sigma_{\rm g}(|\mathbf{k}_\parallel + \mathbf{G}|, \omega)$ for $\mathbf{k}_\parallel$ within the 1BZ of the TMD and the $\mathbf{G}$ vectors running over the reciprocal lattice of this material.

An example of the obtained response function is shown in Fig. S16 for a heterostructure containing $MoSe_2$. We remark that $\mathbf{k}_\parallel$ refers to the optical wave vector, which is associated with the difference between electron and hole wave vectors of the contributing excitons. The response is dominated by nearly direct excitons at $\mathbf{k}_\parallel \approx 0$ as well as weaker features corresponding to $\Gamma Q_{\rm TMD}$ excitons away from that region.

S9. First-principles nonlocal optical response of TMD monolayers

The exciton-dominated nonlocal optical conductivities of monolayer TMDs constitute a central ingredient in our theory. We perform first-principles calculations in the framework of many-body perturbation theory to appropriately describe the excited-state properties of the considered materials. The ground-state wave functions and one-electron eigenenergies are obtained by performing density-functional-theory (DFT) calculations with the Perdew–Burke–Ernzerhof (PBE) [32] exchange–correlation functional as implemented in the Quantum Espresso code [33]. In addition, the spin-orbit interaction is included using optimized norm-conserving pseudopotentials [34, 35] with a kinetic energy cutoff of 80 Ry. We employ the Coulomb-cutoff technique [36] at the edges of the unit cells in the out-of-plane direction with a vacuum layer of 37 atomic units.

Using the DFT output, we perform Bethe–Salpeter-equation (BSE) calculations within the YAMBO code [37, 38]. The G_0W_0 and plasmon-pole approximations [39, 40] are employed to correct the Kohn–Sham eigenenergies. In Fig. S17, we present the so-obtained quasiparticle electronic band dispersions for the TMDs under consideration. The

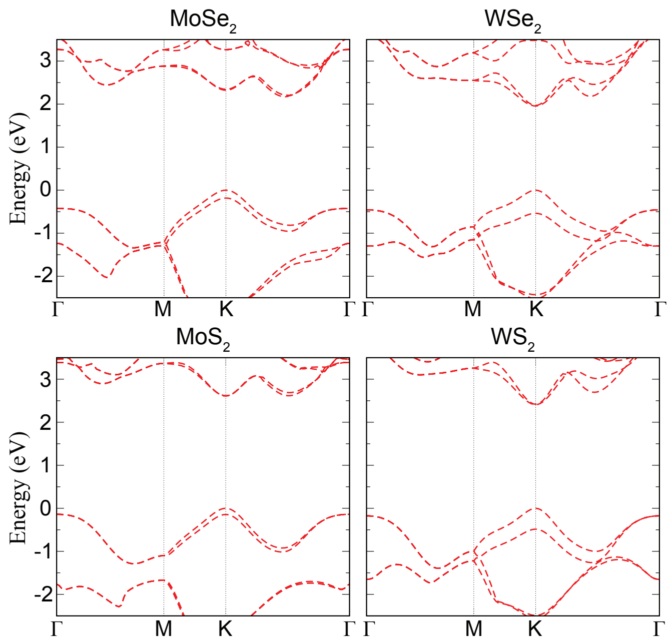

FIG. S17. **Electronic bands of TMD monolayers.** We show DFT calculations for $MoSe_2$, WSe_2, MoS_2, and WS_2, obtained within the G_0W_0 approximation.

direct electronic band gap at the K point is found to be 2.32, 1.95, 2.61, and 2.41 eV for $MoSe_2$, WSe_2, MoS_2, and WS_2, respectively. We solve the BSE [41, 42] to calculate the eigenenergies and eigenvectors of the excitons supported by these materials, from which we obtain the dielectric function. More precisely, we write the BSE as

$$(\varepsilon_{c\mathbf{q}-\mathbf{k}_\parallel} - \varepsilon_{v\mathbf{q}}) A^j_{vc\mathbf{q}\mathbf{k}_\parallel} + \hbar^{-1} \sum_{v'c'\mathbf{q}'} \langle v\mathbf{q}; c\mathbf{q}-\mathbf{k}_\parallel | \mathcal{K}^{\mathrm{eh}} | v'\mathbf{q}'; c'\mathbf{q}'-\mathbf{k}_\parallel \rangle A^j_{v'c'\mathbf{q}'\mathbf{k}_\parallel} = \varepsilon^j_{\mathbf{k}_\parallel} A^j_{vc\mathbf{q}\mathbf{k}_\parallel}, \tag{S37}$$

where a combination of the index j and the in-plane wave vector $\mathbf{k}_\parallel$ are used to label each exciton state, $\hbar\varepsilon^j_{\mathbf{k}_\parallel}$ is the corresponding exciton energy, $\hbar\varepsilon_{v\mathbf{q}}$ and $\hbar\varepsilon_{c\mathbf{q}-\mathbf{k}_\parallel}$ denote valence- and conduction-band quasiparticle energies obtained from G_0W_0 simulations, $|v\mathbf{q}; c\mathbf{q}-\mathbf{k}_\parallel\rangle$ are pure electron–hole excitonic states, $A^j_{vc\mathbf{q}\mathbf{k}_\parallel}$ are the exciton eigenvector coefficients, $\mathcal{K}^{\mathrm{eh}}$ is the electron–hole interaction kernel, and $\mathbf{q}$ are 3D one-electron wave vectors. The generalized oscillator strengths of an excitonic transition can be defined as $\Omega^j_{\mathbf{k}_\parallel\mathbf{G}} = \sum_{vc\mathbf{q}} A^j_{vc\mathbf{q}\mathbf{k}_\parallel} \langle v\mathbf{q}| \mathrm{e}^{-\mathrm{i}(\mathbf{k}_\parallel+\mathbf{G})\cdot\mathbf{R}} | c\mathbf{q}-\mathbf{k}_\parallel\rangle$, from which we calculate the non-interacting susceptibility as

$$\chi^0_{\mathbf{G}\mathbf{G}'}(\mathbf{k}_\parallel,\omega) = \frac{f_s e^2 t}{N_{\mathbf{q}} V \hbar} \sum_j \frac{\Omega^{j*}_{\mathbf{k}_\parallel\mathbf{G}} \Omega^j_{\mathbf{k}_\parallel\mathbf{G}'}}{\omega - \varepsilon^j_{\mathbf{k}_\parallel} + i\gamma}. \tag{S38}$$

Here, we set $f_s = 1$ (2) for spin-polarized (-unpolarized) calculations, $N_{\mathbf{q}}$ is the number of $\mathbf{q}$ points in the 1BZ, V denotes the 3D unit-cell volume, and we multiply by the out-of-plane lattice constant of the simulation unit cell t to obtain a 2D surface response function.

For the two-step tunneling process, we describe direct excitonic transitions using the dipole matrix elements $\langle v\mathbf{q}|\mathrm{e}^{-\mathrm{i}\mathbf{k}_\parallel\cdot\mathbf{R}}|c\mathbf{q}-\mathbf{k}_\parallel\rangle$, calculated from the YAMBO code for small values of $k_\parallel$, in which the in-plane response is isotropic. To obtain an optical spectrum comparable to experimental results, we use a dense and uniform grid of electron wave vectors $\mathbf{q}$ of size 72×72×1 in the G_0W_0 and BSE calculations. We ignore umklapp processes (i.e., we only use the $\mathbf{G} = \mathbf{G}' = 0$ element of the $\chi^0_{\mathbf{G}\mathbf{G}'}$ matrix of components labeled by the 2D reciprocal lattice vectors $\mathbf{G}$ and $\mathbf{G}'$ [37]). Transitions between the four highest-valence and the four lowest-conduction bands are incorporated in the BSE calculations. We employ the Scalable Library for Eigenvalue Problem Computations (SLEPC) [43] to obtain detailed information about each BSE eigenmode and validate our results with the full Lanczos–Haydock solution [44]. Since the matrix that we need to diagonalize is very large, we only extract the BSE eigenmodes for transitions below 3 eV. The 2D conductivities $\sigma_{\mathrm{TMD}}(k_\parallel,\omega)$ of the studied TMD monolayers are obtained from the 2D non-interacting

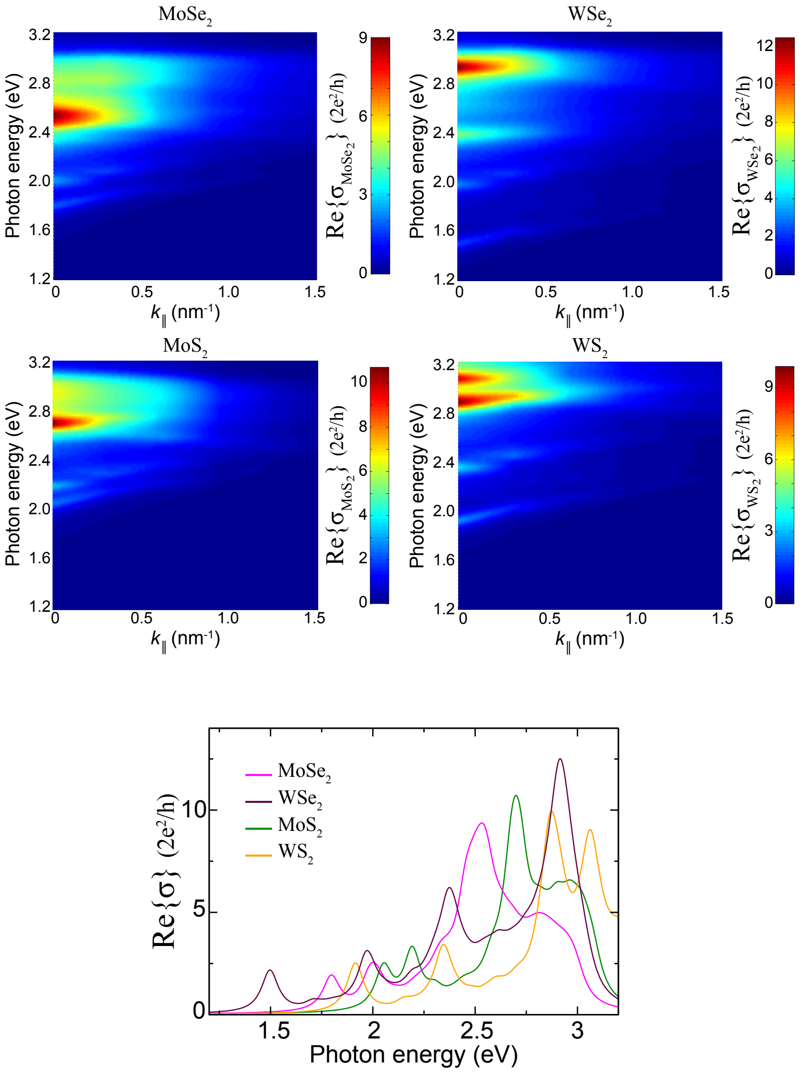

FIG. S18. **Nonlocal conductivity of TMD monolayers.** We plot the calculated real part of the 2D conductivities of $MoSe_2$, WSe_2, MoS_2, and WS_2 monolayers, expressed in units of $2e^2/h$, for relatively low values of the in-plane wave vector $k_\parallel$ and ignoring umklapp processes. The lower panel shows $\mathrm{Re}\{\sigma_{\mathrm{TMD}}(k_\parallel,\omega)\}$ in the $k_\parallel \to 0$ limit.

susceptibility by using the relation $\sigma_{\mathrm{TMD}}(k_\parallel,\omega) = \mathrm{i}\,\omega\chi^0_{00}(k_\parallel,\omega)/k_\parallel^2$. The calculated conductivities are presented in Fig. S18 as a function of photon energy and parallel wave vector.

In the one-step tunneling process, we consider indirect excitonic transitions by calculating the matrix elements $\langle c\mathbf{q}-\mathbf{k}_\parallel|\mathrm{e}^{\mathrm{i}(\mathbf{k}_\parallel+\mathbf{G})\cdot\mathbf{r}}|v\mathbf{q}\rangle$ for $\mathbf{k}_\parallel$ running over the full 1BZ. We also include the first six neighboring Brouillin zones corresponding to the six smallest non-vanishing reciprocal lattice vectors $\mathbf{G}$. To reduce the increased computational burden for such a large set of $\mathbf{k}_\parallel$ and $\mathbf{G}$ vectors, we decrease the $\mathbf{q}$-point grid size to 30×30×1 and do not include spin-orbit coupling in this part of our calculations. In addition, since we focus on momentum-transfer-induced changes in the excitonic spectra, we solve the BSE only for the degenerate highest-valence and lowest-conduction bands. We present examples of the so-obtained conductivity of $MoSe_2$ in Fig. S19, showing features associated with indirect excitons of high momentum within the 1BZ in optical wave vector space $\mathbf{k}_\parallel$.

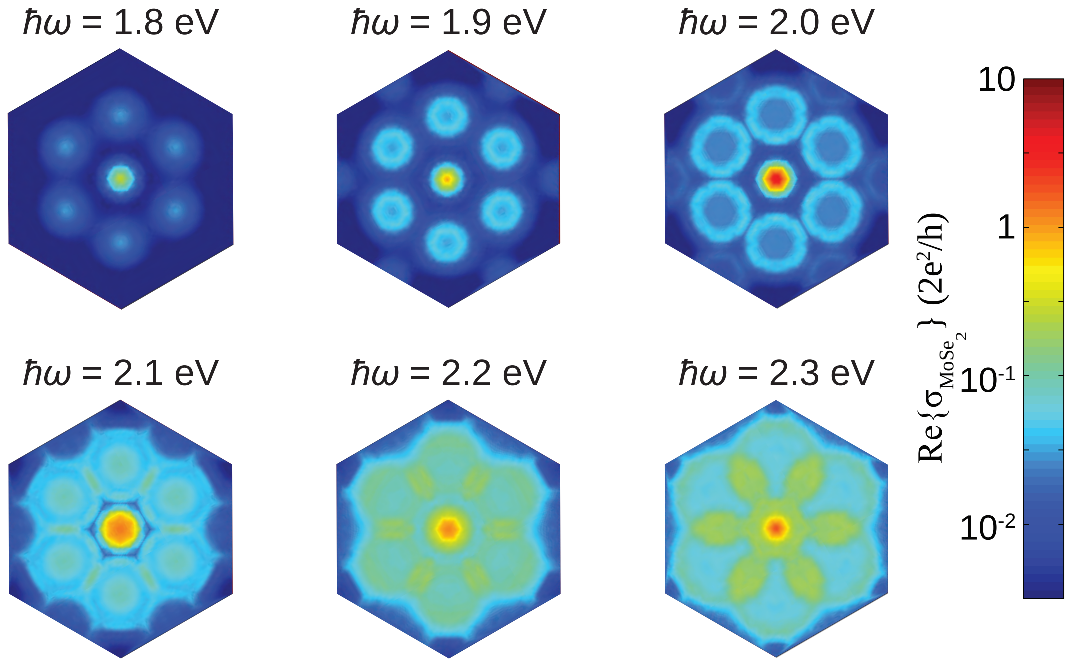

FIG. S19. **Nonlocal response within the 1BZ of $MoSe_2$.** We plot $\mathrm{Re}\{\sigma_{\mathrm{TMD},00}(\mathbf{k}_\parallel, \omega)\}$ (i.e., the real part of the optical conductivity for $\mathbf{G} = \mathbf{G}' = 0$, see Secs. S8 and S9) within the 1BZ of $MoSe_2$ at different photon energies $\hbar\omega$.

[1] M. Parzefall, A. Szabó, T. Taniguchi, K. Watanabe, M. Luisier, and L. Novotny, Light from van der waals quantum tunneling devices, Nat. Commun. **10**, 292 (2019).
[2] F. Joucken, L. Henrard, and J. Lagoute, Electronic properties of chemically doped graphene, Phys. Rev. Materials **3**, 1 (2019).
[3] Y. Zhang, V. W. Brar, F. Wang, C. Girit, Y. Yayon, M. Panlasigui, A. Zettl, and M. F. Crommie, Giant phonon-induced conductance in scanning tunnelling spectroscopy of gate-tunable graphene, Nat. Phys. **4**, 627 (2008).
[4] U. Chandni, K. Watanabe, T. Taniguchi, and J. P. Eisenstein, Evidence for defect-mediated tunneling in hexagonal boron nitride-based junctions, Nano Lett. **15**, 7329 (2015).
[5] K. He, N. Kumar, L. Zhao, Z. Wang, K. F. Mak, H. Zhao, and J. Shan, Tightly bound excitons in monolayer WSe_2, Phys. Rev. Lett. **113**, 026803 (2014).
[6] Y.-N. Xu and W. Y. Ching, Calculation of ground-state and optical properties of boron in the hexagonal, cubic, and wurtzite structures, Phys. Rev. B **44**, 7787 (1991).
[7] M. Mohr, J. Maultzsch, E. Dobardžić, S. Reich, I. Milošević, M. Damnjanović, A. Bosak, M. Krisch, and C. Thomsen, Phonon dispersion of graphite by inelastic x-ray scattering, Phys. Rev. B **76**, 035439 (2007).
[8] R. Senga, K. Suenaga, P. Barone, S. Morishita, F. Mauri, and T. Pichler, Position and momentum mapping of vibrations in graphene nanostructures in the electron microscope, Nature **573**, 247 (2019).
[9] R. Wallauer, R. Perea-Causin, L. Mñster, S. Zajusch, S. Brem, J. Güdde, K. Tanimura, K.-Q. Lin, R. Huber, E. Malic, and U. Höfer, Momentum-resolved observation of exciton formation dynamics in monolayer WS_2, Nano Lett. **21**, 5867 (2021).
[10] Y. Li, A. Chernikov, X. Zhang, A. Rigosi, H. M. Hill, A. M. van der Zande, D. A. Chenet, E.-M. Shih, J. Hone, and T. F. Heinz, Measurement of the optical dielectric function of monolayer transition-metal dichalcogenides: MoS_2, $MoSe_2$, WS_2, and WSe_2, Phys. Rev. B **90**, 205422 (2014).
[11] A. Chernikov, T. C. Berkelbach, H. M. Hill, A. Rigosi, Y. Li, O. B. Aslan, D. R. Reichman, M. S. Hybertsen, and T. F. Heinz, Exciton binding energy and nonhydrogenic rydberg series in monolayer WS_2, Phys. Rev. Lett. **113**, 076802 (2014).
[12] M. Goryca, J. Li, A. V. Stier, T. Taniguchi, K. Watanabe, E. Courtade, S. Shree, C. Robert, B. Urbaszek, X. Marie, and S. A. Crooker, Revealing exciton masses and dielectric properties of monolayer semiconductors with high magnetic fields, Nat. Commun. **10**, 4172 (2019).
[13] A. T. Hanbicki, M. Currie, G. Kioseoglou, A. L. Friedman, and B. T. Jonker, Measurement of high exciton binding energy in the monolayer transition-metal dichalcogenides WS_2 and WSe_2, Sol. State Commun. **203**, 16 (2015).
[14] C. Zhang, Y. Chen, A. Johnson, M.-Y. Li, L.-J. Li, P. C. Mende, R. M. Feenstra, and C.-K. Shih, Probing critical point energies of transition metal dichalcogenides: Surprising indirect gap of single layer WSe_2, Nano Lett. **15**, 6494 (2015).
[15] C. Robert, M. A. Semina, F. Cadiz, M. Manca, E. Courtade, T. Taniguchi, K. Watanabe, H. Cai, S. Tongay, B. Lassagne, P. Renucci, T. Amand, X. Marie, M. M. Glazov, and B. Urbaszek, Optical spectroscopy of excited exciton states in MoS_2 monolayers in van der waals heterostructures, Phys. Rev. Materials **2**, 011001 (2018).
[16] S. Brem, A. Ekman, D. Christiansen, F. Katsch, M. Selig, C. Robert, X. Marie, B. Urbaszek, A. Knorr, and E. Malic, Phonon-assisted photoluminescence from indirect excitons in monolayers of transition-metal dichalcogenides, Nano Lett. **20**, 2849 (2020).
[17] A. V. Stier, N. P. Wilson, K. A. Velizhanin, J. Kono, X. Xu, and S. A. Crooker, Magnetooptics of exciton rydberg states in a monolayer semiconductor, Phys. Rev. Lett. **120**, 057405 (2018).
[18] C. M. Chow, H. Yu, A. M. Jones, J. R. Schaibley, M. Koehler, D. G. Mandrus, R. Merlin, W. Yao, and X. Xu, Quantum corrections in nanoplasmonics: shape, scale, and material, npj 2D Mater. Appl. **1**, 33 (2017).
[19] B. Han, C. Robert, E. Courtade, M. Manca, S. Shree, T. Amand, P. Renucci, T. Taniguchi, K. Watanabe, X. Marie, L. E. Golub, M. M. Glazov, and B. Urbaszek, Exciton states in monolayer $MoSe_2$ and $MoTe_2$ probed by upconversion spectroscopy, Phys. Rev. X **8**, 031073 (2018).
[20] J. Horng, T. Stroucken, L. Zhang, E. Y. Paik, H. Deng, and S. W. Koch, Observation of interlayer excitons in $MoSe_2$ single crystals, Phys. Rev. B **97**, 241404 (2018).
[21] A. H. Castro Neto, F. Guinea, N. M. R. Peres, K. S. Novoselov, and A. K. Geim, The electronic properties of graphene, Rev. Mod. Phys. **81**, 109 (2009).
[22] M. Abramowitz and I. A. Stegun, *Handbook of Mathematical Functions* (Dover, New York, 1972).
[23] F. J. García de Abajo, Optical excitations in electron microscopy, Rev. Mod. Phys. **82**, 209 (2010).
[24] D. Pines and P. Noziéres, *The Theory of Quantum Liquids* (W. A. Benjamin, Inc., New York, 1966).
[25] S. de Vega and F. J. García de Abajo, Plasmon generation through electron tunneling in graphene, ACS Photonics **4**, 2367 (2017).
[26] A. Rodríguez Echarri, J. D. Cox, and F. J. García de Abajo, Quantum effects in the acoustic plasmons of atomically-thin heterostructures, Optica **6**, 630 (2019).
[27] B. Wunsch, T. Stauber, F. Sols, and F. Guinea, Dynamical polarization of graphene at finite doping, New. J. Phys. **8**, 318 (2006).
[28] R. Geick, C. H. Perry, and G. Rupprecht, Normal modes in hexagonal boron nitride, Phys. Rev. **146**, 543 (1966).
[29] F. J. García de Abajo, Graphene plasmonics: challenges and opportunities, ACS Photonics **1**, 135 (2014).
[30] F. J. García de Abajo, Nonlocal effects in the plasmons of strongly interacting nanoparticles, dimers, and waveguides, J. Phys. Chem. C **112**, 17983 (2008).
[31] R. H. Ritchie and A. L. Marusak, The surface plasmon dispersion relation for an electron gas, Surf. Sci. **4**, 234 (1966).

[32] J. P. Perdew, K. Burke, and M. Ernzerhof, Generalized gradient approximation made simple, Phys. Rev. Lett. **77**, 3865 (1996).

[33] P. Giannozzi, S. Baroni, N. Bonini, M. Calandra, R. Car, C. Cavazzoni, D. Ceresoli, G. L. Chiarotti, M. Cococcioni, I. Dabo, A. D. Corso, S. d. Gironcoli, S. Fabris, G. Fratesi, R. Gebauer, U. Gerstmann, C. Gougoussis, A. Kokalj, M. Lazzeri, L. Martin-Samos, N. Marzari, F. Mauri, R. Mazzarello, S. Paolini, A. Pasquarello, L. Paulatto, C. Sbraccia, S. Scandolo, G. Sclauzero, A. P. Seitsonen, A. Smogunov, P. Umari, and R. M. Wentzcovitch, QUANTUM ESPRESSO: a modular and open-source software project for quantum simulations of materials, J. Phys. Condens. Matter **21**, 395502 (2009).

[34] D. R. Hamann, Optimized norm-conserving vanderbilt pseudopotentials, Phys. Rev. B **88**, 085117 (2013).

[35] M. Schlipf and F. Gygi, Optimization algorithm for the generation of ONCV pseudopotentials, Comput. Phys. Commun. **196**, 36 (2015).

[36] A. C. Rozzi, D. Varsano, A. Marini, E. K. U. Gross, and A. Rubio, Exact Coulomb cutoff technique for supercell calculations, Phys. Rev. B **73**, 205119 (2006).

[37] A. Marini, C. Hogan, M. Grüning, and D. Varsano, Yambo: an *ab initio* tool for excited state calculations, Comput. Phys. Commun. **180**, 1392 (2009).

[38] D. Sangalli, A. Ferretti, H. Miranda, C. Attaccalite, I. Marri, E. Cannuccia, P. Melo, M. Marsili, F. Paleari, A. Marrazzo, G. Prandini, P. Bonfa, M. O. Atambo, F. Affinito, M. Palummo, A. Molina-Sanchez, C. Hogan, M. Grüning, D. Varsano, and A. Marini, Many-body perturbation theory calculations using the yambo code, J. Phys. Condens. Matter **31**, 325902 (2019).

[39] L. Hedin, New method for calculating the one-particle Green's function with application to the electron-gas problem, Phys. Rev. **139**, A796 (1965).

[40] G. Onida, L. Reining, and A. Rubio, Electronic excitations: density-functional versus many-body Green's-function approaches, Rev. Mod. Phys. **74**, 601 (2002).

[41] M. Rohlfing and S. G. Louie, Electron-hole excitations and optical spectra from first principles, Phys. Rev. B **62**, 4927 (2000).

[42] M. Palummo, O. Pulci, R. D. Sole, A. Marini, P. Hahn, W. G. Schmidt, and F. Bechstedt, The Bethe-Salpeter equation: a first-principles approach for calculating surface optical spectra, J. Phys. Condens. Matter **16**, S4313 (2004).

[43] V. Hernandez, J. E. Roman, , and V. Vidal, SLEPc: A scalable and flexible toolkit for the solution of eigenvalue problems, ACM Trans. Math. Softw. **31**, 351 (2005).

[44] R. Haydock, The recursive solution of the Schrödinger equation, Solid State Phys. **35**, 215 (1980).
